# Activity of Birinapant, a SMAC Mimetic Compound, Alone or in Combination in NSCLCs With Different Mutations

**DOI:** 10.3389/fonc.2020.532292

**Published:** 2020-10-22

**Authors:** Marika Colombo, Mirko Marabese, Giulia Vargiu, Massimo Broggini, Elisa Caiola

**Affiliations:** Laboratory of Molecular Pharmacology, Istituto di Ricerche Farmacologiche Mario Negri IRCCS, Milan, Italy

**Keywords:** non-small-cell lung cancer, liver kinase B1, KRAS, drug library *in vitro* screening, SMAC mimetic compounds, 3D culture (three-dimensional spheroids), combination therapeutics, Ralimetinib

## Abstract

Liver kinase B1 (*LKB1/STK11*) is the second tumor suppressor gene most frequently mutated in non-small-cell lung cancer (NSCLC) and its activity is impaired in about half KRAS-mutated NSCLCs. Nowadays, no effective therapies are available for patients having these mutations. To highlight new vulnerabilities of this subgroup of tumors exploitable to design specific therapies we screened an US FDA-approved drug library using an isogenic system of wild-type (WT) or deleted LKB1. Among eight hit compounds, Birinapant, an inhibitor of the Inhibitor of Apoptosis Proteins (IAPs), was the most active compound in LKB1-deleted clone only compared to its LKB1 WT counterpart. We validated the Birinapant cells response and its mechanism of action to be dependent on LKB1 deletion. Indeed, we demonstrated the ability of this compound to induce apoptosis, through activation of caspases in the LKB1-deleted clone only. Expanding our results, we found that the presence of *KRAS* mutations could mediate Birinapant resistance in a panel of NSCLC cell lines. The combination of Birinapant with Ralimetinib, inhibitor of p38α, restores the sensitivity of LKB1- and KRAS-mutated cell lines to the IAP inhibitor Birinapant. Our study shows how the use of Birinapant could be a viable therapeutic option for patients with LKB1-mutated NSCLCs. In addition, combination of Birinapant and a KRAS pathway inhibitor, as Ralimetinib, could be useful for patients with LKB1 and KRAS-mutated NSCLC.

## Introduction

Non-small-cell lung cancer (NSCLC) is the leading cause of cancer-related death worldwide (1). In the recent decades, with the discovery of the molecular heterogeneity and oncogene addiction of some NSCLC subtypes, the use of targeted therapies and immunotherapy has improved the outcomes for patients affected by these malignancies (2). Nowadays, in spite of this progress, some mutations frequently present in NSCLCs remain untargetable and the available therapies seem to be not very effective (2, 3). Among these, NSCLC mutated in *Liver kinase B1* (*LKB1/STK11)* gene represent one-third of the cases and *LKB1/STK11* is considered the third most commonly mutated gene in NSCLC adenocarcinomas, after *TP53* and *KRAS* (4). *LKB1* mutations were found in about 50% of KRAS-mutated NSCLC and it was demonstrated that the co-occurrence of *LKB1/KRAS* mutations significantly increases the tumor burden, mediated by increased resistance to classical anticancer and immunotherapeutic drugs thus corresponding with poor prognosis for patients carrying these alterations (5, 6). Moreover, mutations in *LKB1* is mutually exclusive with mutations in those genes for which a targeted therapy already exists. LKB1 is a master kinase that, acting on AMPK-mTOR pathway, regulates different cellular processes as cell metabolism, cell polarity, growth and autophagy (7). Mutations in this gene almost invariably lead to protein loss of function that reflects in a series of cellular abnormalities (8).

Birinapant is a SMAC mimetic compound and an IAP inhibitor (9). Similar to the endogenous SMAC protein, Birinapant is able to bind IAPs promoting their degradation. In particular, it binds with high affinity to the cellular IAP 1 (c-IAP1) and with a lesser extent to the cellular IAP 2 (c-IAP2) and XIAP (10). IAPs belong to the class of proteins that inhibit the apoptotic process. Indeed, in normal cell conditions, they block the extrinsic apoptotic pathway and promote cell survival and cell growth (11, 12).

Ralimetinib is a selective molecule able to inhibit α and β isoforms of p38 mitogen-activated protein kinase (MAPK), in an ATP-competitive way (13). P38 MAPK belongs to MAPK family, which also includes JNK and ERK (14), and it is downstream the MAPKKK proteins, as KRAS protein. P38 MAPK protein phosphorylates multiple substrates in response to external stimuli. Inhibition of this protein decreases prosurvival, proangiogenic, and proinflammatory soluble factors (15).

In the present study, after an FDA-approved drug library screening, we analyzed the activity of Birinapant alone, or in combination with Ralimetinib, in LKB1-mutated NSCLC cell lines.

## Materials and Methods

### 2D Cell Culture and Treatments

The NSCLC cell lines used (H1299, H520, H1975, H2009, H358, LU99, H727, H460, H2030, A549, H23) were obtained from American Type Culture Collection (ATCC) and RIKEN BRC cell bank. They were grown in RPMI1640 (Gibco) with the addition of 10% fetal bovine serum (FBS) (Euroclone) and 2 mM L-Glutamine (Gibco). Two LKB1-deleted clones H1299-LKB1 KO 1 and 2 were derived from H1299 through the Crispr-Cas9 technique, as previously described (16). They were maintained in selection by adding 3 µg/ml of Puromycin to the medium. The NCI-H1299 cells were also genetically manipulated to generate the KRAS G12C mutated (K) and LKB1WT and KRAS G12C-mutated and LKB1-deleted (KL) clones (17–20). For the K and KL clones, 500 µg/ml of Geneticin (G418) were added to the medium. Cell lines were routinely tested for mycoplasma contamination by polymerase chain reaction (PCR), and authenticated with the PowerPlex 16 HS System (Promega) every 6 months by comparing the short tandem repeat (STR) profiles to those deposited in the ATCC and/or in the German Collection of Microorganisms and Cell Cultures (DSMZ) databases.

The day of the treatment, dimethyl sulfoxide (DMSO) stock solutions of all the drugs used (10 mM) were diluted in complete medium at the desired concentrations with a final DMSO concentration of 0.05% for single treatment or 0.15% in combination treatment. In all the cytotoxicity experiments, either single or combination treatments, cells were continuously treated for 72 h.

Cell viability assays were performed independently. For the MTS cell viability assay, MTS was added to each well. Then, plates were incubated at 37°C for about 3 h and the absorbance at 490 nm was read using plate-reading instrument (GloMax discover, Promega). For the CellTiter-Glo viability assay, a volume of CellTiter-Glo reagent equal to the volume present in each well was added and luminescence was read through GloMax instrument. Finally, the sulforhodamine B assay was performed following the manufacturer’s instructions and the absorbance was measured at 560 nm.

For each experiment, starting from the absorbance/luminescence values, the mean of at least six biological replicates and the percentage of cell viability (where the 100% of viability were control-treated samples values) were calculated for each dose. The average of at least three independent experiments was then plotted in dose-response curves. The concentration that inhibits 50% of cell viability (IC_50_) was calculated with PRISM software.

### 3D Spheroids Culture and Treatments

Procedures involving animals were conducted in conformity with the following laws, regulations, and policies governing the care and use of laboratory animals: Italian Governing Law (D. lg 26/2014; authorization no.19/2008-A issued 6 March 2008 by the Ministry of Health); Mario Negri Institutional Regulations and Policies providing internal authorization for persons conducting animal experiments (Quality Management System Certificate: UNI EN ISO 9001:2008, reg. no. 6121); An institutional review board and the Italian Ministry of Health approved the *in vivo* experiments performed (project authorization #9F5F5.69.EXT.37).

Three dimensional spheroids models were derived from excised H1299 and H1299-LKB1 KO xenografts, obtained by subcutaneously injecting the cell lines in nude mice. When the tumor weight was about 1 mg, the mice were euthanized with CO_2_, then, tumors were excised, rinsed with saline solution, mechanically minced and incubated in a flask at 37°C with collagenase. After 30 min, all the flask content was filtered, the tumor mass was recovered and another cycle with collagenase was performed for 60 min. Successively, after filtration, the tumor mass was transferred into a 50 ml falcon where it was resuspended in 10 ml of wash buffer (**Supplementary Material**) and incubated at room temperature for 20 min. The supernatant was removed and the passage was repeated until the solution became clear. After the last wash, the pellet was spun down in 10 ml of wash buffer. The pellet was resuspended in wash buffer and counted with Neubauer chamber. Cells, at a density of 20000 cells/ml, were then resuspended in 50 µl/well of Basement Membrane Extract (BME, RGF BME, Type 2 PathClear, CULTREX) and seeded in 24-well plate. Once BME was solidified, 500 µl of culture medium (**Supplementary Material**) was added. Spheroids formed in about 1 week and were subsequently subcultured once a week. The procedure of subculture consisted in mechanical detachment of BME with spheroids from the substrate and trypsin addition (TrypLE Express, GIBCO) to favor the disruption of the 3D aggregates. The suspension was then incubated at 37°C for 5 min under mild shaking and trypsin activity was stopped by adding cold basal medium (**Supplementary Material**). Single cells were resuspended in BME and plated in 24-well plates. After BME solidification, warm culture medium was added. Spheroids were replaced with fresh stocks from liquid nitrogen after 4 to 5 months of culture.

To perform cytotoxicity experiments, 3D spheroids were mechanically detached from the 24-well plate and spheroid-derived single cells were then counted by Neubauer chamber and resuspended in an appropriated volume of BME to obtain 100,000 cells/ml as final concentration. They were then seeded in white 96-well plates, 10 µl of BME per well, and 50 µl of culture medium was added to each well. Four days after seeding, they were treated and 72 h after treatment start, CellTiter glo assay was performed, as previously described.

### FDA-Approved Drug Library Screening

The FDA approved drug library (Z208828, Selleckchem) is a collection of 1,443 inhibitors belonging to different classes like oncology, anti-inflammation, immunology, neuropsychiatry, analgesia and so on. The library comprises some drugs already approved by FDA and some undergoing clinical trials (21). The compounds were dissolved in DMSO or water at a concentration of 10 mM. Original stock solutions of the library compounds were then diluted in water to a final concentration of 100 μM for each compound.

To perform the screening, cells were detached from flasks by trypsin-EDTA, resuspended at the desired concentration in RPMI1640 medium plus Penicillin and Streptomycin (Pen/Strep, Gibco) and seeded in a volume of 76 µl/well in 384-wells plates by automatic liquid handling (epMotion 5075, Eppendorf). The next day, the plates were treated with the FDA-approved drug library by an automatic liquid handling. Four µl of each drug were transferred to 384-well plates, thus reaching a final concentration of 5 µM for each drug and 0.05% for DMSO. Medium (80 μl/well) was used as a blank. Negative control was composed of 4 μl of H_2_O or 4 μl of H_2_O + DMSO 0.05% without drug treatment. Finally, a positive control group was composed of adding 4 μl of a drug known to be active only in LKB1-deleted clone. After 72 h of continuous treatment, the cytotoxicity of each compound was evaluated with MTS cell viability assay, as previously described. For each cell line, the absorbance of drug-treated cells (T) was normalized to control-treated cells (C), thus obtaining the T/C ratio.

### Western Blot Molecular Analysis

Cells were seeded in petri dishes and, after 48 h, they were treated with drug at the desired concentration. Pellets were collected 24 and 48 h after treatment start. To prepare the pellets, cells were washed twice with ice-cold PBS and then mechanically detached from the plates with scrapers. The suspension was then centrifuged and the pellet resuspended in lysis buffer and incubated on ice for 1 h to permit cells lysis. Successively, insoluble cellular debris were pelleted at 10,000 rpm for 15 min at 4°C and the total protein amount in the supernatant was recovered. An aliquot was used to determine protein concentration at 595 nm at the spectrophotometer Ultrospec 2100 pro (Amersham Bioscences). Protein concentration was obtained using a BSA calibration curve.

Thirty µg of protein total extracts were separated according to their molecular weight with an electrophoretic run in denaturing conditions at about 100V. Then, proteins were transferred on activated PVDF membrane (Millipore) for 2 h at 60V. PVDF membrane was colored with Red Ponceau dye (Sigma) to verify the presence of proteins.

The proteins of interest were detected by exposing PVDF membranes overnight at 4°C to protein-specific primary antibodies diluted in 5% BSA-TBS-T or non-fat dry milk-TBS-T. The next day, the membrane was exposed to the secondary antibodies labelled with horseradish-peroxidase. After several washings, the horseradish-peroxidase substrate (ECL Western Blotting Detection, Amersham-Life Science) was added and the signal revealed through Odyssey Fc instrument (LI-COR). C-IAP1, LKB1, and PARP primary antibodies were purchased from Cell Signaling while XIAP, Caspase-3, Actin, Ran, and Lamin B from Santa Cruz Biothecnology. The anti-mouse and anti-rabbit secondary antibodies were purchased from Biorad whereas the anti-goat secondary antibody was purchased from Santa Cruz Biotechnology.

### Realtime-Glo Annexin V Apoptosis Assay

Cells were seeded in white 96-well plates and treated with the drug at the desired concentrations after 24 h. The Realtime-Glo Annexin V detection reagent was added to all wells (the detection reagent was prepared following the datasheet instructions). Plates were maintained at 37°C and luminescence was read at different time points: 0, 24, 48, and 72 h after treatment start. At each concentration and time point, luminescence data were normalized to blank values and the average of six biological replicates was calculated. Control-treated cells were used as reference samples. Results were plotted as histograms, which represented the mean of at least three independent experiments.

### Caspase-Glo 3/7 Assay

Cells were seeded in white 96-well plates and, after 24 h, treated with the drug at the desired concentrations. Seventy-two hours after treatment, the Caspase Glo reagent was then added to all wells (the reagent was prepared following the datasheet instructions). Plates were incubated at 37°C and, after 1 h, luminescence was read. Data were analyzed as for Annexin V assay and statistical analysis was performed with PRISM software.

### Statistical Analysis

The statistical analyses for each experiment were performed through GraphPad Prism 7.01 software (GraphPad Software, San Diego California USA, www.graphpad.com). The different tests used are reported in the legends of the figures. Differences with a p-value < 0.05 were considered statistically significant.

## Results

### FDA-Approved Drug Library Screening and Independent Validation of the Hit Compounds

**Figure 1:**
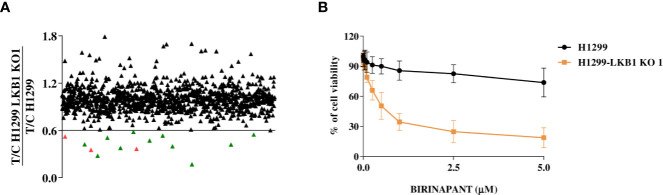
**(A)**, Distribution of the FDA-approved drug library’s compounds according to their different activity in H1299 and H1299 LKB1 KO 1 cells. Y-axis refers to H1299-LKB1 KO 1 T/C and H1299 T/C ratio. Each single triangle represents a compound. The eleven hit compounds are below the chosen cut-off value of 0.6 and they are colored in green. The three excluded compounds are in red. **(B)** Dose-response curves of H1299 and H1299-LKB1 KO 1 isogenic system treated with increasing concentrations of Birinapant. The response to the drug was evaluated with MTS assay. The average of three independent experiments is reported. Statistical analysis was carried out through two-way ANOVA and Bonferroni post-test for multiple comparisons and it is reported in **Supplementary Table 2**.

To find new vulnerabilities of LKB1-mutated NSCLCs, potentially exploitable to design new therapies, we performed a high throughput screening with an FDA-approved drug library. We used NCI-H1299 cell line (LKB1 WT) and a LKB1-deleted clone (H1299-LKB1 KO 1) previously obtained with Crispr-Cas9 technique (16) from H1299. To select compounds more active on LKB1-deleted clone than on the parental cell line, for each compound of the library, the ratio between H1299-LKB1 KO 1 T/C and H1299 T/C was calculated and a cut-off of 0.6 was established (**Supplementary Table 1**). This cut-off permitted to select compounds able to induce at least 40% more cell killing in LKB1-deleted clone than in the WT cell line. Fourteen compounds have achieved the cut-off value but three of them were excluded from hit compounds because of their high toxicity in both cell lines (**Figure 1A**). Among hits, two MEK inhibitors, three antimetabolites, a SYK inhibitor and an ALDH inhibitor, an HMG-CoA reductase inhibitor, an antiseptic, an antibacterial and an IAP inhibitor were present (**Table 1**).

**Table 1:** Table summarizing the H1299 and H1299-LKB1 KO 1 clone (here reported as LKB1 KO 1) IC_50_ of the eleven hit compounds from the drug library screening.

DRUG	CLASS	H1299 IC_50_ (μM)	LKB1 KO 1 IC_50_ (μM)	IC_50_ H1299 / IC_50_ LKB1 KO 1
Birinapant	IAP inhibitor	>5	0.527 (0.466–0.596)	≥9.48
6-Mercaptopurine	Antimetabolite	>5	0.212 (0.184–0.244)	≥2.36
Clofarabine	Antimetabolite	0.717 (0.559–0.954)	0.332 (0.289–0.385)	2.16
Floxuridine	Antimetabolite	0.106 (0.065–0.178)	0.054 (0.038–0.789)	1.95
Pitavastatin Calcium	HMG-CoA reductase inhibitor	3.114 (1.681–13.159)	1.687 (1.028–3.877)	1.84
Fostamatinib	SYK inhibitor	4.380 (3.904–5.156)	2.660 (2.506–2.819)	1.65
Chloroxine	Antibacterial	3.399 (3.138–3.682)	3.100 (2.878–3.326)	1.10
Disulfiram	ADLH-inhibitor	0.241 (0.136–0.412)	0.289 (0.159–0.514)	0.83
Chlorhexidine HCl	Antiseptic	0.747 (0.626–0.876)	1.942 (1.792–2.101)	0.39
Cobimetinib	MEK inhibitor	>4	>4	–
Pimasertib	MEK inhibitor	>4	>4	–

For each compound the drug class, the IC_50_ in the two cell lines and the ratio between the IC_50_ in the H1299 and H1299-LKB1 KO 1 clone are indicated.

The activity of selected drugs was confirmed by generating, for each compound, a complete dose-response curve on both H1299 and H1299-LKB1 KO 1 cell lines and by calculating the IC_50,_ where it was possible. In addition, the ratio between H1299 IC_50_ and H1299-LKB1 KO 1 IC_50_ was calculated. Eight compounds, out of eleven, confirmed a higher cytotoxicity on the LKB1-deleted clone compared to the parental cell line (**Table 1**, **Supplementary Figure 1**). The antiseptic and antibacterial drugs showed an opposite behavior compared to the screening, while the ALDH inhibitor did not show significant differences between the two cell lines. Although the H1299-LKB1 KO 1 showed a higher sensitivity to MEK inhibitors than the LKB1 WT cell line, drug concentrations chosen for these inhibitors were too low to reach the IC_50_ in both cell lines. Birinapant, an IAP inhibitor, gave the best different responses: H1299-LKB1 KO 1 showed a significant sensitivity to this drug compared to the parental cell line and only for the LKB1-mutated clone we were able to calculate the IC_50_ (0.53 µM; CI: 0.47–0.60 µM) (**Figure 1B**, **Table 1**, and **Supplementary Table 2**).

Therefore, the study continued characterizing the different response observed with this drug.

### Analysis of Birinapant Activity With Different Cell Viability Assays and in 3D Models

**Figure 2:**
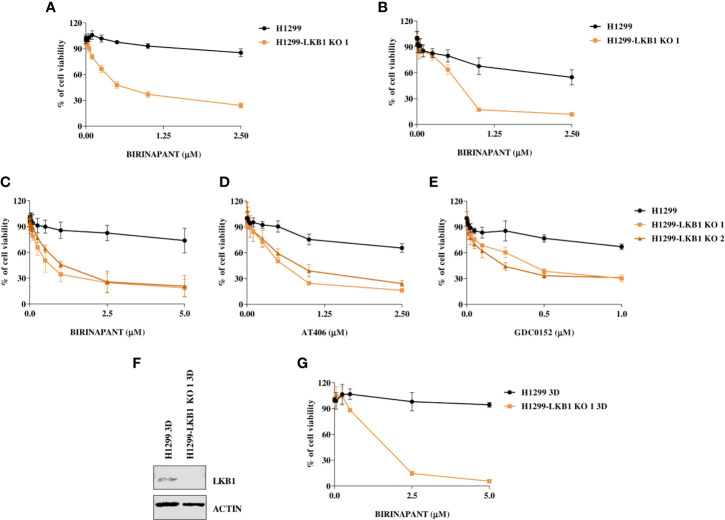
**(A, B)** Evaluation of the H1299 isogenic system response to Birinapant treatment through different cell viability assays: **(A)** CellTiter-Glo viability assay and **(B)** Sulforhodamine B assay. Dose-response curves were generated by treating the H1299 isogenic system with increasing concentrations of Birinapant. The average of three independent experiments is reported. Statistical analysis was carried out through two-way ANOVA and Bonferroni post-test for multiple comparisons and it is reported in **Supplementary Table 3**. **(C–E)** Dose-response curves of H1299 and two H1299-LKB1 KO clones treated with increasing concentrations of **(C)** Birinapant, **(D)** AT406, and **(E)** GDC0152. The response to the drug was evaluated with the MTS assay. The average of three independent experiments is reported. Statistical analysis was carried out through two-way ANOVA and Bonferroni post-test for multiple comparisons and it is reported in **Supplementary Table 4**. **(F)** Western Blot analysis of LKB1 expression levels in H1299 and H1299-LKB1 KO 1 spheroids (3D). Actin was used as a loading control. **(G)** Dose-response curves of H1299 and H1299-LKB1 KO 1 spheroids (3D) treated with increasing concentrations of Birinapant. The response to the drug was evaluated with the CellTiter-Glo viability assay. The average of three independent experiments is reported. Statistical analysis was carried out through two-way ANOVA and Bonferroni post-test for multiple comparisons and it is reported in **Supplementary Table 5**.

Birinapant activity was further validated on our isogenic system by two additional cell viability assays. The use of CellTiter-Glo and the sulforodamine B assay confirmed the high sensitivity of the LKB1-deleted clone to Birinapant (IC_50_ 0.52 μM; CI: 0.46–0.58 μM and 0.53 μM; CI: 0.41–0.67 μM, respectively), while the parental cell line remained resistant, with an IC_50_ not calculable for the first assay and equal to 2.58 μM (CI: 2.0–3.4 μM) for the second (**Figures 2A, B** **and** **Supplementary Table 3**). Moreover, the Birinapant activity was confirmed in another independent H1299-derived clone (H1299-LKB1 KO 2) previously obtained with the Crispr-Cas9 technique (**Figure 2C**, **Supplementary Table 4**) (16).

In order to verify that Birinapant activity was correlated to its specific mechanism of action as IAP inhibitor, we treated the cell lines with increasing doses of other two IAP inhibitors, AT406 and GDC0152. For each drug, it was possible to calculate the IC_50_ in both the two LKB1-deleted clones, whereas the drug concentrations used did not permit to reach the 50% of viability inhibition in the LKB1 WT cell line (**Figures 2D, E** **and** **Supplementary Table 4**).

Finally, we increased the complexity of our cellular model: 3D spheroids expressing or not LKB1 were generated (**Figure 2F**) and treated with Birinapant. Even in this model, it was possible to calculate the IC_50_ just for the clone lacking LKB1 (1.3 µM; CI: 0.8–2.0 µM) while the WT cells resulted resistant (**Figure 2G** and **Supplementary Table 5**).

### Analysis of Apoptosis at Different Levels After Birinapant Treatment

**Figure 3:**
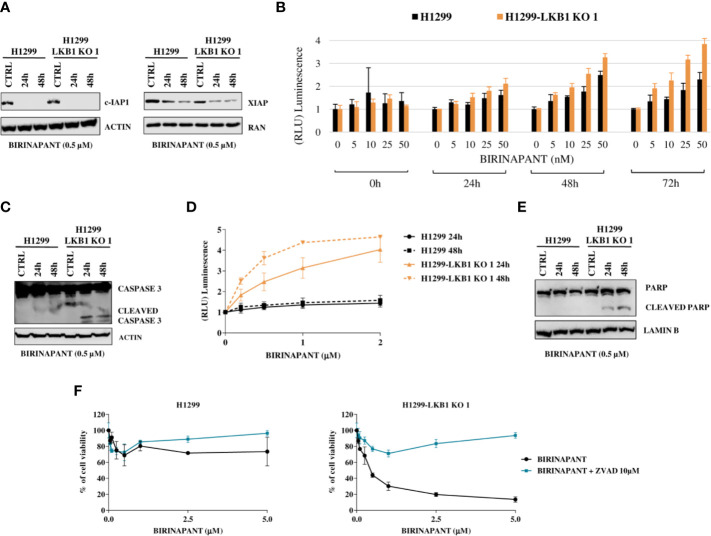
**(A)** Western Blot analysis of c-IAP and XIAP levels after Birinapant treatment, at different time points. Actin and Ran were used as loading controls. **(B)** RealTime-Glo Annexin V assay on H1299 and H1299-LKB1 KO 1 cell lines treated with Birinapant 5, 10, 25, and 50 nM. Annexin V exposure was followed 0, 24, 48, and 72 h from treatment start. The average of three biological replicates with standard deviations are reported. Statistical analysis was carried out through two-way ANOVA and Bonferroni post-test for multiple comparisons and it is reported in **Supplementary Table 6**. **(C)** Analysis of caspase-3 cleavage in H1299 and H1299-LKB1 KO 1. The two cell lines were treated with Birinapant 0.5 μM and protein levels were evaluated 24 and 48 h after treatment start. Actin was used as loading control. **(D)** Evaluation of caspase-3/7 activity in H1299 isogenic system after treatment with different concentrations of Birinapant, 24 and 48 h after treatment start with Caspase-Glo 3/7 assay. The average of two independent experiments is reported. The statistical analysis was carried out through two-way ANOVA and Bonferroni post-test for multiple comparisons and it is reported in **Supplementary Table 7**. **(E)** Analysis of PARP cleavage in H1299 isogenic system after 24 and 48 h from Birinapant treatment start. Lamin B was used as loading control. **(F)** Dose-response curves of H1299 and H1299-LKB1 KO 1 cell lines treated with increasing concentrations of Birinapant, alone or in combination with ZVAD 10 μM. The response to the drugs was evaluated with MTS assay. The average of three independent experiments is reported. Statistical analysis was carried out through two-way ANOVA and Bonferroni post-test for multiple comparisons and it is reported in **Supplementary Table 8**.

We first excluded that the differences in the sensitivity of H1299 and H1299-LKB1 KO 1 cells to the drug were due to differences in the achievement of its targets. We analyzed the expression of c-IAP1 and XIAP, the two main Birinapant targets, after 24 and 48 h from treatment start with a sub-toxic concentration of 0.5 µM. In both cell lines, at the two time points considered, the drug reached its targets but, while the c-IAP1 was completely degraded, the XIAP levels were only (again equally in both cell lines) downregulated (**Figure 3A**).

Being an IAP inhibitor, Birinapant exerts its cytotoxic activity by induction of apoptosis (9), so we analyzed this process at different levels. We evaluated the phosphatidylserine (PS) exposure on the outer leaflet of cell membrane, a signal of induction of apoptosis (22), by detecting Annexin V binding to it. H1299 and H1299-LKB1 KO 1 cells were treated with increasing concentrations of Birinapant and Annexin V binding was measured at different time points. We observed a higher PS exposure in H1299-LKB1 KO 1 clone compared to H1299, after 24 h of treatment. The differences in Annexin V levels between the two cell lines became more marked at 48 and 72 h (**Figure 3B** **and** **Supplementary Table 6**).

Then, we studied the effectors of apoptosis, Caspase 3 and 7. We observed the cleaved and active form of the Caspase 3 enzyme after treatment with a sub-toxic dose of Birinapant (0.5 µM), just in H1299-LKB1 KO cell line, while in H1299 only the uncleaved, inactive form of Caspase 3 was present (**Figure 3C**). Further evidence of apoptosis activation only in H1299-LKB1 KO 1 sensitive clone was given by the observation of Caspase 3/7 activity, at both 24 and 48 h from treatment. In the WT cell line, no activity of effector caspases at the two time points considered was detected (**Figure 3D** and **Supplementary Table 7**). In addition, Birinapant treatment induced cleavage of PARP, a substrate of active caspases, once again just in H1299-LKB1 KO 1 cells (**Figure 3E**).

To corroborate these data with a different approach, we treated cells with a combination of Birinapant and ZVAD, a pan caspases inhibitor. As expected, the co-treatment completely restored the resistance to Birinapant in H1299-LKB1 KO 1 clone while the same treatment in H1299 parental cell line did not change the viability of cells. Indeed, while the LKB1-deleted clone displayed an IC_50_ of about 0.5 μM, when treated with Birinapant alone, it was not possible to calculate this parameter when ZVAD was added to the treatment (**Figure 3F** and **Supplementary Table 8**).

### Analysis of Birinapant Activity in NSCLC Cell Lines With Different *LKB1* Status

**Figure 4:**
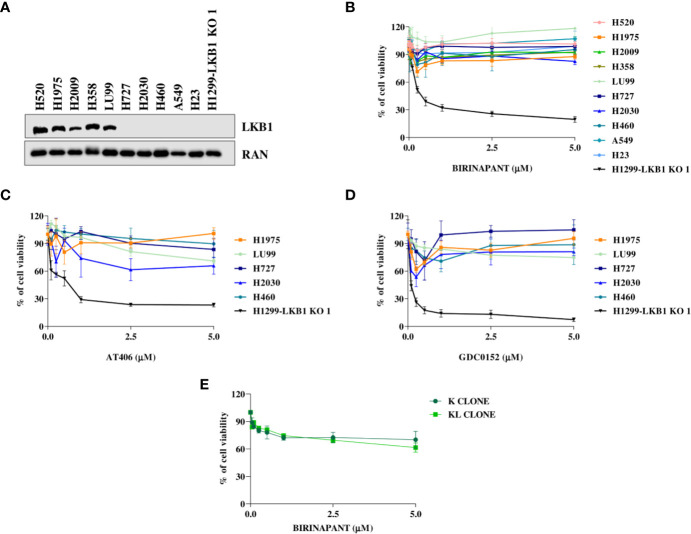
**(A)** Western Blot analysis of LKB1 expression levels in NSCLC cell lines. Ran was used as loading control. **(B–D)** Dose-response curves of NSCLC cell lines treated with increasing concentrations of **(B)** Birinapant, **(C)** AT406, and **(D)** GDC0152. The response to the drugs was evaluated with MTS assay. The average of three independent experiments is reported. **(E)** Dose-response curves of H1299-derived clones, K, and KL, treated with increasing concentrations of Birinapant. The response to the drugs was evaluated with MTS assay. The average of three independent experiments is reported. Statistical analysis was carried out through two-way ANOVA and Bonferroni post-test for multiple comparisons. Data were not reported because no differences were found among all the compared groups.

In order to strengthen our previous results, we expanded the study to a panel of NSCLC cell lines WT or naturally mutated in LKB1 (**Table 2**). As already reported in literature, all the inactivating mutations found in *LKB1* gene invariably lead to protein loss (**Figure 4A**) (7). All the cell lines chosen, together with the H1299-LKB1 KO 1 clone, used as positive control of treatment efficacy, were treated with Birinapant and dose-response curves were plotted. As shown in **Figure 4B**, all the cell lines were resistant to the compound (IC_50_ > 5 μM), independently from their *LKB1* mutational status. These findings were also confirmed by treating a representative panel of cell lines, with increasing concentrations of two other IAP inhibitors previously tested, AT406 (**Figure 4C**) and GDC0152 (**Figure 4D**). Realizing that all LKB1-mutated cell lines in this panel also harbored activating *KRAS* mutations (**Table 2**) we hypothesized that these alterations could impede, in some way, Birinapant action and justify the resistance of LKB1-mutated NSCLC cell lines.

**Table 2:** *LKB1* and *KRAS* mutational status in NSCLC cell lines.

CELL LINES	LKB1	KRAS	REFs
H520	WT	WT	(23)
H1975	WT	WT	(23)
H2009	WT	G12A	(23)
H358	WT	G12C	(23)
LU99	WT	G12C	(24)
H727	Q302P	G12V	(23)
H460	LOSS (Hom Q37*)	Q61H	(23)
H2030	LOSS (Hom E317*)	G12C	(23)
A549	LOSS (Hom Q37*)	G12S	(23)
H23	LOSS (Hom W322*)	G12C	(23)

REFs references of the mutational status reported in the table.

To assess this hypothesis, we used an *ad hoc* isogenic cell system composed by K clone (KRAS G12C/LKB1 WT) and its derived KL clone (KRAS G12C/LKB1-deleted). The K clone was previously obtained starting from H1299 cell line by transfecting KRAS-G12C containing vector, then *LKB1* was disrupted through Crispr-Cas9 technique, thus generating KL clone (17–20). Both clones were resistant to Birinapant as indicated by IC_50_ values higher than 5 μM (**Figure 4E**).

### Combination of Birinapant and Ralimetinib in Other KRAS-LKB1 Co-Mutated Cell Lines

**Figure 5:**
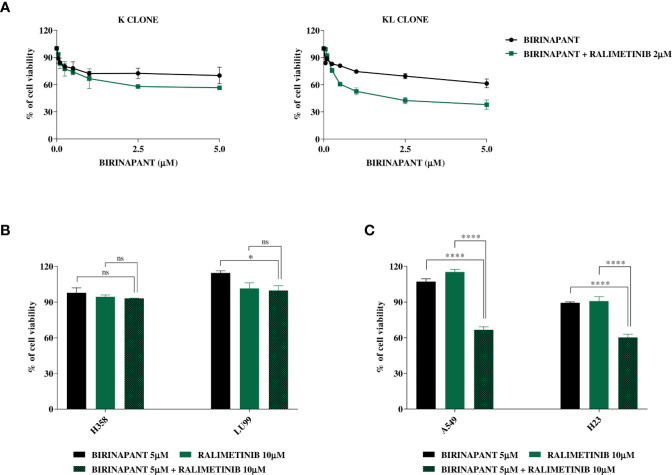
**(A)** Dose-response curves of K and KL clones treated with increasing concentrations of Birinapant, alone or in combination with Ralimetinib 2 μM. The response to the drugs was evaluated with MTS assay. The average of three independent experiments is reported. Statistical analysis was carried out through two-way ANOVA and Bonferroni post-test for multiple comparisons and it is reported in **Supplementary Table 9**. **(B)** Histograms of KRAS mutated/LKB1 WT cell lines, H358 and LU99, treated with Birinapant 5 μM, Ralimetinib 10 μM or the combination of the two drugs. The response to the drugs was evaluated with MTS assay. The average of three independent experiments is reported. Statistical analysis was carried out through one-way ANOVA and Bonferroni post-test for multiple comparisons and it is reported in the graphs. *p < 0.05, ns: not statistically significant. **(C)** Histograms of KRAS mutated/LKB1 deleted cell lines, A549 and H23, treated with Birinapant 5 μM, Ralimetinib 10 μM or the combination of the two drugs. The response to the drugs was evaluated with MTS assay. The average of three independent experiments is reported. Statistical analysis was carried out through one-way ANOVA and Bonferroni post-test for multiple comparisons and it is reported in the graphs. ****p < 0.0001. Percentage of cell viability of single treated samples was calculated reporting its value to the control-treated sample, considered 100% viable. While, for combination treatment, Ralimetinib treated sample was considered as 100% of viability hence the combination cell viability was normalized on the Ralimetinib cell viability. In this way, the minimum effect of Ralimetinib on cell viability is nulled and the potency of Ralimetinib in sensitizing cells to Birinapant is highlighted.

Knowing that the unique difference between KL and H1299-LKB1 KO 1 clones is the activating mutation in *KRAS*, we suggested that the inhibition of proteins belonging to pathways downstream of KRAS could restore the sensitivity to Birinapant in KL clone. Therefore, we treated K and KL clones with increasing doses of Birinapant and a sub-toxic dose of some KRAS downstream protein inhibitors: ERK inhibitor, MEK inhibitor, B-RAF inhibitor, AKT inhibitor and p38α inhibitor (data not shown). Among them, only the combination of Birinapant and Ralimetinib (2 μM), the p38α inhibitor, restored the sensitivity in KL clone, with an IC_50_ of 1.55 μM (CI: 1.24–1.99 μM), while the K clone remained resistant (IC_50_ > 5 μM) (**Figure 5A** and **Supplementary Table 9**).

Having confirmed that, in our *ad hoc* isogenic system, the inhibition of a KRAS downstream protein with Ralimetinib restored the sensitivity to Birinapant in the LKB1-deleted KRAS mutated clone, we tried to expand this result to other LKB1 and KRAS mutated cell lines of our previous panel. We chose two KRAS mutated and LKB1 WT cell lines, the H358 and the LU99, and other two naturally mutated both in KRAS and LKB1, the A549 and the H23. All these cell lines were treated with single sub-toxic doses of Birinapant or Ralimetinib and with the combination of the two drugs. The combination treatment did not have a significant impact on cell viability in the LKB1 WT cell lines (**Figure 5B**), while it significantly decreased cell viability in both the LKB1-mutated cell lines (**Figure 5C**).

## Discussion

Mutations in *STK11*, the second tumor suppressor gene most frequently mutated in NSCLCs (4), lead to loss of LKB1 protein expression which precludes the possibility to directly targeting the cancer-associated, mutated product. Moreover, the frequent co-presence, in NSCLCs, of *LKB1* and *KRAS* mutations (25) is associated with resistance to the classical anticancer drugs and immunotherapy (5). In addition, the mutual exclusivity of *LKB1* mutations with other targetable mutated genes, such as EGFR and ALK (5), pose great challenges on how to treat patients affected by these tumors. Therefore, highlighting new vulnerabilities of LKB1-mutated NSCLC tumors potentially exploitable to design new therapies is urgently needed. In order to achieve this goal, we have screened an FDA-approved drug library on a NSCLC cell line LKB1 WT and its LKB1-deleted clone, previously obtained in our laboratory with Crispr-Cas9 technique. The FDA-approved library used comprises some drugs already approved by FDA and others under evaluation in clinical trials (21). The screening of FDA-approved library is an interesting technique already used in different fields by researchers (26, 27). Indeed, by examining all the drug classes on the same cellular model it is possible to find new applications for an old drug and, hence, introduce an already existing therapy to a new disease, the so called drug repurposing (28). In addition, drug repurposing allows rapid clinical impact and patient benefit at reduced cost and time requirements than *de novo* drug development as a result of the availability of bioactivity and safety data from clinical trials for each compound used in the screening (21). In our study, the screening and the consequent validation revealed that eight compounds were more active on the LKB1-deleted clone compared to the parental cell line. As already reported in the literature, the LKB1-deleted clone resulted sensitive to the three different MEK inhibitors included in the library (29, 30). The most active compound on LKB1-deleted clone compared to the LKB1 WT cell line was Birinapant, a phase II SMAC mimetic or IAP inhibitor compound. Our results indicate that Birinapant, as a representative compound of IAP inhibitors, inhibited c-IAP1 and XIAP in both cell lines, but just in the LKB1-deleted clone it induced apoptosis through caspase activation. Studies in literature showed that degradation or inhibition of IAPs by Birinapant does not necessarily translate in sensitivity to the drug (31). In our isogenic system, the unique difference between the cell lines is the deletion in LKB1, so we identify a potential role of this protein in determining sensitivity to IAP inhibitors.

In order to enhance the translational impact of our results, we chose to shift from this isogenic system to different LKB1 WT or naturally mutated NSCLC cell lines. All the cell lines tested were resistant to Birinapant, independently from *LKB1* mutations. Due to the fact that all LKB1-deleted cell lines tested in these experiments were also KRAS-mutated, we hypothesized that the latter mutation could constitutively activate downstream pathways to interfere with the sensitivity of LKB1-deleted cell lines to IAP inhibitors. To investigate the hypothesized contribution of *KRAS* mutations in the resistance to Birinapant, we combined subtoxic doses of the IAP inhibitor to different KRAS downstream protein inhibitors. Among them, the combination of Birinapant and Ralimetinib, a p38α inhibitor (13, 14), was able to restore the sensitivity of Birinapant in KRAS- and LKB1-mutated cell lines. Our data are in line with those present in literature where it was shown that targeting p38α, overcomes resistance to Birinapant in primary acute myeloid leukemia (32).

In conclusion, our results highlighted a potential new strategy to specifically treat LKB1-deleted tumors. Pending the verification of our results in LKB1-mutated *in vivo* systems, the use of Birinapant could be a viable therapeutic option for patients with LKB1-mutated NSCLCs, where co-existing alterations which can interfere with Birinapant activity (i.e. *KRAS* activating mutations) have not been found. Moreover, combination of Birinapant and Ralimetinib could be also useful for that number of patients with LKB1- and KRAS-mutated NSCLC, for whom, no targeted therapies are available yet, although the recent introduction of KRAS G12C specific inhibitors could make KRAS druggable (33). Because the results observed with Ralimetinib were based on the assumption that it interferes with KRAS signaling, our data would suggest that a combination of Birinapant and KRAS specific inhibitors, (at least for those patients harboring G12C mutation) could be a further valuable strategy. Finally, considering that Birinapant has been already adopted in phase I-II clinical trials (NCT03803774, NCT01828346, and NCT01681368) (34), the new therapy could be quickly translated to the clinic.

## Data Availability Statement

The datasets generated for this study are available on request to the corresponding author.

## Ethics Statement

The animal study was reviewed and approved by The Mario Negri institutional review board and the Italian Ministry of Health.

## Author Contributions

EC, MM, and MB contributed to the conception and design of the study. MC, MM, GV, and EC organized and performed the experiments. MB and EC performed the statistical analysis. MC and EC wrote the first draft of the manuscript. MC, MM, GV, MB, and EC wrote sections of the manuscript. All authors contributed to the article and approved the submitted version.

## Funding

This work was supported by Fondazione CARIPLO “Biomedical research conducted by young researchers (2018-0372) grant to EC.

## Conflict of Interest

The authors declare that the research was conducted in the absence of any commercial or financial relationships that could be construed as a potential conflict of interest.

## References

[1] Planchard D Popat S Kerr K Novello S Smit EF Faivre-Finn C Metastatic non-small cell lung cancer: ESMO Clinical Practice Guidelines for diagnosis, treatment and follow-up. Ann Oncol (2018) 29(Suppl 4):iv192–237. 10.1093/annonc/mdy275 30285222

[2] Boolell V Alamgeer M Watkins D Ganju V The Evolution of Therapies in Non-Small Cell Lung Cancer. Cancers (2015) 7(3):1815–46. 10.3390/cancers7030864 PMC4586797 26371045

[3] Baxevanos P Mountzios G Novel chemotherapy regimens for advanced lung cancer: have we reached a plateau? Ann Trans Med (2018) 6(8):139–9. 10.21037/atm.2018.04.04 PMC5952027 29862228

[4] Ding L Getz G Wheeler DA Mardis ER McLellan MD Cibulskis K Somatic mutations affect key pathways in lung adenocarcinoma. Nature (2008) 455(7216):1069–75. 10.1038/nature07423 PMC2694412 18948947

[5] Facchinetti F Bluthgen MV Tergemina-Clain G Faivre L Pignon J-P Planchard D LKB1/STK11 mutations in non-small cell lung cancer patients: Descriptive analysis and prognostic value. Lung Cancer (2017) 112:62–8. 10.1016/j.lungcan.2017.08.002 29191602

[6] Ji H Ramsey MR Hayes DN Fan C McNamara K Kozlowski P LKB1 modulates lung cancer differentiation and metastasis. Nature (2007) 448(7155):807–10. 10.1038/nature06030 17676035

[7] Zhou W Zhang J Marcus AI LKB1 Tumor Suppressor: Therapeutic Opportunities Knock when LKB1 Is Inactivated. Genes Dis (2014) 1(1):64–74. 10.1016/j.gendis.2014.06.002 25679014 PMC4323096

[8] Momcilovic M Shackelford DB Targeting LKB1 in cancer - exposing and exploiting vulnerabilities. Br J Cancer (2015) 113(4):574–84. 10.1038/bjc.2015.261 PMC4647688 26196184

[9] Benetatos CA Mitsuuchi Y Burns JM Neiman EM Condon SM Yu G Birinapant (TL32711), a bivalent SMAC mimetic, targets TRAF2-associated cIAPs, abrogates TNF-induced NF-κB activation, and is active in patient-derived xenograft models. Mol Cancer Ther (2014) 13(4):867–79. 10.1158/1535-7163.MCT-13-0798 24563541

[10] Amaravadi RK Schilder RJ Martin LP Levin M Graham MA Weng DE A Phase I Study of the SMAC-Mimetic Birinapant in Adults with Refractory Solid Tumors or Lymphoma. Mol Cancer Ther (2015) 14(11):2569–75. 10.1158/1535-7163.MCT-15-0475 26333381

[11] Silke J Meier P Inhibitor of apoptosis (IAP) proteins-modulators of cell death and inflammation. Cold Spring Harb Perspect Biol (2013) 5(2). 10.1101/cshperspect.a008730 PMC3552501 23378585

[12] Fulda S Vucic D Targeting IAP proteins for therapeutic intervention in cancer. Nat Rev Drug Discovery (2012) 11(2):109–24. 10.1038/nrd3627 22293567

[13] Campbell RM Anderson BD Brooks NA Brooks HB Chan EM De Dios A Characterization of LY2228820 Dimesylate, a Potent and Selective Inhibitor of p38 MAPK with Antitumor Activity. Mol Cancer Ther (2014) 13(2):364–74. 10.1158/1535-7163.MCT-13-0513 24356814

[14] Manning G Whyte DB Martinez R Hunter T Sudarsanam S The protein kinase complement of the human genome. Science (2002) 298(5600):1912–34. 10.1126/science.1075762 12471243

[15] Patnaik A Haluska P Tolcher AW Erlichman C Papadopoulos KP Lensing JL A First-in-Human Phase I Study of the Oral p38 MAPK Inhibitor, Ralimetinib (LY2228820 Dimesylate), in Patients with Advanced Cancer. Clin Cancer Res (2016) 22(5):1095–102. 10.1158/1078-0432.CCR-16-0645 26581242

[16] Caiola E Iezzi A Tomanelli M Bonaldi E Scagliotti A Colombo M LKB1 Deficiency Renders NSCLC Cells Sensitive to ERK Inhibitors. J Thorac Oncol (2019) 15(3):360–70. 10.1016/j.jtho.2019.10.009 31634668

[17] Brunelli L Caiola E Marabese M Broggini M Pastorelli R Comparative metabolomics profiling of isogenic KRAS wild type and mutant NSCLC cells in vitro and in vivo. Sci Rep (2016) 6(1). 10.1038/srep28398 PMC4916601 27329432

[18] Caiola E Brunelli L Marabese M Broggini M Lupi M Pastorelli R Different metabolic responses to PI3K inhibition in NSCLC cells harboring wild-type and G12C mutant KRAS. Oncotarget (2016) 7(32):51462–72. 10.18632/oncotarget.9849 PMC5239488 27283493

[19] Caiola E Salles D Frapolli R Lupi M Rotella G Ronchi A Base excision repair-mediated resistance to cisplatin in KRAS(G12C) mutant NSCLC cells. Oncotarget (2015) 6(30):30072–87. 10.18632/oncotarget.5019 PMC4745782 26353932

[20] Garassino MC Marabese M Rusconi P Rulli E Martelli O Farina G Different types of K-Ras mutations could affect drug sensitivity and tumour behaviour in non-small-cell lung cancer. Ann Oncol (2011) 22(1):235–7. 10.1093/annonc/mdq680 21169473

[21] https://www.selleckchem.com/.

[22] Hassan M Watari H AbuAlmaaty A Ohba Y Sakuragi N Apoptosis and molecular targeting therapy in cancer. BioMed Res Int (2014) 2014:150845. 10.1155/2014/150845 25013758 PMC4075070

[23] https://cancer.sanger.ac.uk/cell_lines.

[24] Takata M Chikumi H Miyake N Adachi K Kanamori Y Yamasaki A Lack of AKT activation in lung cancer cells with EGFR mutation is a novel marker of cetuximab sensitivity. Cancer Biol Ther (2012) 13(6):369–78. 10.4161/cbt.19238 22313637

[25] Skoulidis F Byers LA Diao L Papadimitrakopoulou VA Tong P Izzo J Co-occurring Genomic Alterations Define Major Subsets of KRAS-Mutant Lung Adenocarcinoma with Distinct Biology, Immune Profiles, and Therapeutic Vulnerabilities. Cancer Discovery (2015) 5(8):860–77. 10.1158/2159-8290.CD-14-1236 PMC4527963 26069186

[26] Wang S Liu Y Guo J Wang P Zhang L Xiao G Screening of FDA-Approved Drugs for Inhibitors of Japanese Encephalitis Virus Infection. Diamond MS, editor. J Virol (2017) 91(21). 10.1128/JVI.01055-17 PMC5640845 28814523

[27] Guillotin D Austin P Begum R Freitas MO Merve A Brend T Drug-Repositioning Screens Identify Triamterene as a Selective Drug for the Treatment of DNA Mismatch Repair Deficient Cells. Clin Cancer Res (2017) 23(11):2880–90. 10.1158/1078-0432.CCR-16-1216 PMC5457806 27913567

[28] Hirst J Pathak HB Hyter S Pessetto ZY Ly T Graw S Licofelone Enhances the Efficacy of Paclitaxel in Ovarian Cancer by Reversing Drug Resistance and Tumor Stem-like Properties. Cancer Res (2018) 78(15):4370–85. 10.1158/0008-5472.CAN-17-3993 PMC6072598 29891506

[29] Wang Y Li N Jiang W Deng W Ye R Xu C Mutant LKB1 Confers Enhanced Radiosensitization in Combination with Trametinib in KRAS-Mutant Non-Small Cell Lung Cancer. Clin Cancer Res (2018) 24(22):5744–56. 10.1158/1078-0432.CCR-18-1489 30068711

[30] Mahoney CL Choudhury B Davies H Edkins S Greenman C van Haaften G LKB1/KRAS mutant lung cancers constitute a genetic subset of NSCLC with increased sensitivity to MAPK and mTOR signalling inhibition. Br J Cancer (2009) 100(2):370–5. 10.1038/sj.bjc.6604886 PMC2634725 19165201

[31] Krepler C Chunduru SK Halloran MB He X Xiao M Vultur A The novel SMAC mimetic birinapant exhibits potent activity against human melanoma cells. Clin Cancer Res (2013) 19(7):1784–94. 10.1158/1078-0432.CCR-12-2518 PMC3618495 23403634

[32] Lalaoui N Hänggi K Brumatti G Chau D Nguyen N-YN Vasilikos L Targeting p38 or MK2 Enhances the Anti-Leukemic Activity of Smac-Mimetics. Cancer Cell (2016) 29(2):145–58. 10.1016/j.ccell.2016.01.006 26859455

[33] Hallin J Engstrom LD Hargis L Calinisan A Aranda R Briere DM The KRASG12C Inhibitor MRTX849 Provides Insight toward Therapeutic Susceptibility of KRAS-Mutant Cancers in Mouse Models and Patients. Cancer Discovery (2020) 10(1):54–71. 10.1158/2159-8290.CD-19-1167 31658955 PMC6954325

[34] https://clinicaltrials.gov/ct2/home.

