# Supplementary figures and images for: Activity of Birinapant, a SMAC Mimetic Compound, Alone or in Combination in NSCLCs With Different Mutations
Source: Front Oncol. 2020 Oct 22;10:532292. doi: 10.3389/fonc.2020.532292 (PMC7643013; doi:10.3389/fonc.2020.532292)

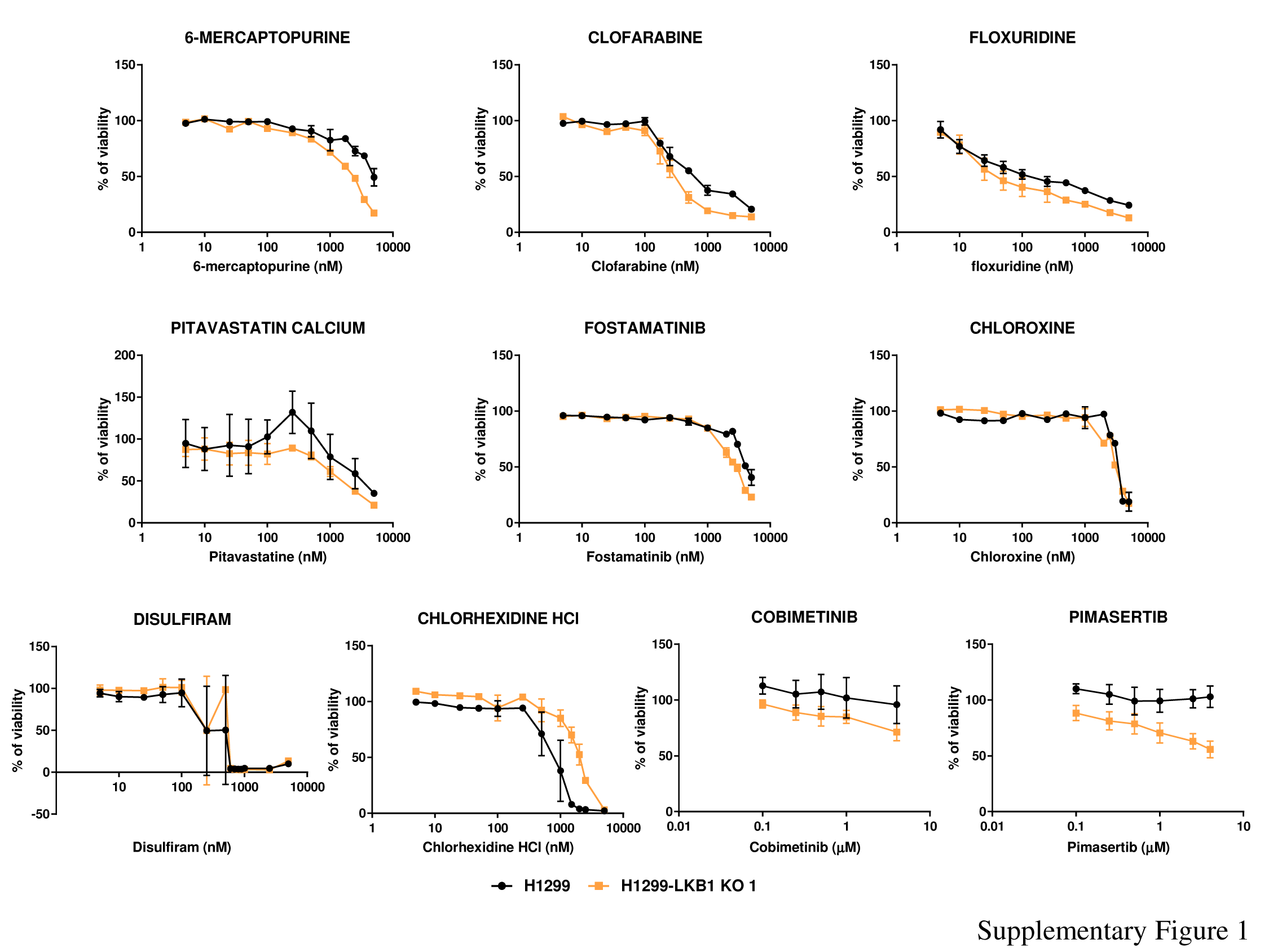

Supplement: Supplementary file 1 [file Image_1.tif]
