# Supplementary material for: Activity of Birinapant, a SMAC Mimetic Compound, Alone or in Combination in NSCLCs With Different Mutations
Source: Front Oncol. 2020 Oct 22;10:532292. doi: 10.3389/fonc.2020.532292 (PMC7643013; doi:10.3389/fonc.2020.532292)
Supplement: Supplementary file 2 [file DataSheet_1.docx]

**Supplementary Material**

**Supplementary Figure Legend**

**Supplementary Figure 1.** Dose-response curves of H1299 and H1299-LKB1 KO 1 cell lines treated with 10 hit drugs at different concentrations. The response to the drugs was evaluated 72 h from the treatment start with the MTS assay. The average of three independent experiments, with standard deviations, are reported.

**Supplementary Materials and methods**

Spheroids wash buffer composition

| **WASH BUFFER** | |
| --- | --- |
| F12 | 1000 mL |
| FBS | 5% |
| Pen/Strep 1% | 10 mL |

Spheroids culture medium composition

| **CULTURE MEDIUM** | |
| --- | --- |
|  | **Final concentration** |
| DMEM:F12 | 1:3 |
| PENICILLIN | 100 units/mL |
| STREPTOMYCIN | 100 µg/mL |
| GlutaMAX | 2 mM |
| HEPES | 1 mM |
| FBS | 5% |
| B27 | 50X (confidential, Thermo Fischer Scientific) |
| N-acetilcysteine | 125 µM |
| EGF | 50 ng/mL |
| R-spondin conditional medium | 10% |
| Wnt conditional medium | 10% |

Spheroids basal medium composition

| **BASAL MEDIUM** | |
| --- | --- |
|  | **Final concentration** |
| DMEM:F12 | 1:3 |
| PENICILLIN | 100 units/mL |
| STREPTOMYCIN | 100 µg/mL |
| GlutaMAX | 2 mM |
| HEPES | 1 mM |

**Supplementary Tables**

**Supplementary Table 1. H1299-LKB1 KO1 T/C, H1299 T/C and H1299-LKB1 KO1 T/C/H1299 T/C ratio of all the 1443 FDA-approved library compounds.** Compounds having the T/C ratio less than 0.6 are considered hits and they are coloured in green. Among them, three compounds were excluded (red) because of their high toxicity in both cell lines at the concentration used in the screening.

| COMPOUNDS | H1299 T/C | H1299-LKB1 KO 1 T/C | $\frac{\mathbf{H1299-LKB1 KO 1 T/C}}{\mathbf{H1299 T/C}}$ |
| --- | --- | --- | --- |
| ABT-263 (Navitoclax) | 1,20 | 1,26 | 1,05 |
| TAK-700 (Orteronel) | 1,12 | 1,21 | 1,08 |
| Melatonin | 1,19 | 1,10 | 0,93 |
| Afatinib (BIBW2992) | 0,38 | 0,35 | 0,93 |
| Imatinib Mesylate (STI571) | 1,32 | 1,14 | 0,86 |
| Sunitinib Malate | 0,73 | 0,68 | 0,94 |
| Masitinib (AB1010) | 1,03 | 1,11 | 1,07 |
| Quisinostat (JNJ-26481585) | 0,05 | 0,05 | 0,91 |
| YM155 (Sepantronium Bromide) | 0,01 | 0,01 | 0,52 |
| Roscovitine (Seliciclib,CYC202) | 0,92 | 0,88 | 0,95 |
| Ritonavir | 1,13 | 1,08 | 0,95 |
| Linifanib (ABT-869) | 0,57 | 0,62 | 1,08 |
| Exemestane | 1,21 | 1,18 | 0,97 |
| Bisoprolol fumarate | 1,04 | 1,03 | 0,99 |
| Bortezomib (PS-341) | 0,03 | 0,01 | 0,36 |
| Lapatinib (GW-572016) Ditosylate | 1,13 | 1,04 | 0,91 |
| Temsirolimus (CCI-779, NSC 683864) | 0,67 | 0,53 | 0,78 |
| GDC-0941 | 1,04 | 0,86 | 0,83 |
| Rucaparib (AG-014699,PF-01367338) | 1,07 | 0,91 | 0,86 |
| Alisertib (MLN8237) | 0,40 | 0,27 | 0,68 |
| Capecitabine | 1,16 | 1,15 | 0,99 |
| Anastrozole | 1,07 | 1,13 | 1,05 |
| Veliparib (ABT-888) | 1,20 | 1,39 | 1,16 |
| Finasteride | 1,11 | 1,01 | 0,92 |
| Tivozanib (AV-951) | 0,67 | 0,69 | 1,04 |
| Bosutinib (SKI-606) | 0,52 | 0,52 | 0,99 |
| Lenalidomide (CC-5013) | 1,23 | 1,18 | 0,97 |
| Vandetanib (ZD6474) | 0,96 | 0,80 | 0,84 |
| Crizotinib (PF-02341066) | 0,35 | 0,25 | 0,72 |
| Vatalanib (PTK787) 2HCl | 1,10 | 0,98 | 0,89 |
| Malotilate | 1,16 | 1,13 | 0,98 |
| CEP-18770 (Delanzomib) | 0,01 | 0,02 | 1,93 |
| Aprepitant | 1,14 | 1,05 | 0,92 |
| Axitinib | 0,98 | 1,08 | 1,10 |
| Irinotecan | 0,54 | 0,38 | 0,70 |
| Doxorubicin (Adriamycin) | 0,15 | 0,27 | 1,76 |
| Cediranib (AZD2171) | 0,99 | 0,88 | 0,88 |
| Panobinostat (LBH589) | 0,04 | 0,02 | 0,67 |
| Vorinostat (SAHA, MK0683) | 0,34 | 0,42 | 1,23 |
| Vismodegib (GDC-0449) | 1,04 | 1,00 | 0,96 |
| Foretinib (GSK1363089) | 0,16 | 0,13 | 0,82 |
| 17-AAG (Tanespimycin) | 0,14 | 0,17 | 1,20 |
| Ganetespib (STA-9090) | 0,10 | 0,11 | 1,09 |
| Bicalutamide | 0,95 | 0,88 | 0,93 |
| Saracatinib (AZD0530) | 0,68 | 0,75 | 1,11 |
| Cladribine | 0,40 | 0,33 | 0,82 |
| Fluorouracil (5-Fluoracil, 5-FU) | 0,26 | 0,42 | 1,62 |
| Dovitinib (TKI-258, CHIR-258) | 0,89 | 0,88 | 0,99 |
| Nilotinib (AMN-107) | 0,85 | 0,93 | 1,09 |
| VX-680 (Tozasertib, MK-0457) | 0,28 | 0,32 | 1,14 |
| Belinostat (PXD101) | 0,06 | 0,04 | 0,67 |
| Triciribine | 0,59 | 0,80 | 1,35 |
| Ivacaftor (VX-770) | 0,95 | 1,11 | 1,16 |
| AT13387 | 0,08 | 0,17 | 2,04 |
| Fulvestrant | 0,88 | 1,12 | 1,27 |
| FG-4592 | 1,04 | 1,31 | 1,26 |
| Decitabine | 0,44 | 0,52 | 1,17 |
| Methotrexate | 0,17 | 0,30 | 1,74 |
| Dasatinib | 0,42 | 0,39 | 0,92 |
| Pazopanib HCl | 0,97 | 0,89 | 0,92 |
| Entinostat (MS-275) | 0,33 | 0,51 | 1,57 |
| Iniparib (BSI-201) | 1,31 | 1,31 | 1,00 |
| Cabozantinib (XL184, BMS-907351) | 0,90 | 0,91 | 1,01 |
| Barasertib (AZD1152-HQPA) | 0,62 | 0,59 | 0,96 |
| Lenvatinib (E7080) | 0,84 | 0,80 | 0,95 |
| Raltitrexed | 0,55 | 0,52 | 0,94 |
| Selumetinib (AZD6244) | 1,09 | 0,93 | 0,85 |
| Dimesna | 1,02 | 1,05 | 1,03 |
| Imiquimod | 1,00 | 1,04 | 1,03 |
| Erlotinib HCl (OSI-744) | 0,99 | 0,81 | 0,81 |
| Rapamycin (Sirolimus) | 0,50 | 0,55 | 1,10 |
| Enzastaurin (LY317615) | 0,84 | 0,83 | 0,99 |
| PCI-24781 (Abexinostat) | 0,28 | 0,26 | 0,95 |
| Everolimus (RAD001) | 0,42 | 0,47 | 1,12 |
| Docetaxel | 0,17 | 0,28 | 1,59 |
| Valproic acid sodium salt (Sodium valproate) | 0,96 | 1,00 | 1,05 |
| Thalidomide | 1,00 | 0,94 | 0,94 |
| Nintedanib (BIBF 1120) | 1,35 | 1,12 | 0,83 |
| Dutasteride | 0,87 | 1,01 | 1,16 |
| Bendamustine HCl | 1,18 | 1,18 | 1,00 |
| Gefitinib (ZD1839) | 1,03 | 1,17 | 1,13 |
| Sorafenib Tosylate | 0,98 | 0,81 | 0,82 |
| Olaparib (AZD2281, Ku-0059436) | 1,02 | 1,10 | 1,08 |
| OSI-906 (Linsitinib) | 0,93 | 0,84 | 0,90 |
| Mocetinostat (MGCD0103) | 0,13 | 0,25 | 1,89 |
| Paclitaxel | 0,13 | 0,15 | 1,20 |
| Regorafenib (BAY 73-4506) | 0,77 | 0,75 | 0,98 |
| CUDC-101 | 0,16 | 0,22 | 1,39 |
| Nelarabine | 1,33 | 1,37 | 1,03 |
| Rigosertib (ON-01910) | 0,09 | 0,13 | 1,42 |
| Ruxolitinib (INCB018424) | 1,08 | 0,95 | 0,89 |
| Raloxifene HCl | 1,41 | 1,32 | 0,94 |
| Agomelatine | 1,26 | 1,22 | 0,97 |
| Posaconazole | 1,16 | 1,27 | 1,09 |
| Adapalene | 1,18 | 1,26 | 1,06 |
| Bumetanide | 1,23 | 1,19 | 0,97 |
| Megestrol Acetate | 1,27 | 1,21 | 0,95 |
| Etomidate | 1,17 | 0,99 | 0,85 |
| Glimepiride | 1,18 | 1,00 | 0,85 |
| Bleomycin Sulfate | 0,18 | 0,16 | 0,89 |
| Epothilone B (EPO906, Patupilone) | 0,17 | 0,28 | 1,69 |
| Isotretinoin | 1,21 | 1,20 | 0,99 |
| Fludarabine Phosphate | 1,13 | 0,96 | 0,85 |
| Amuvatinib (MP-470) | 1,06 | 0,75 | 0,71 |
| Prasugrel | 1,34 | 1,12 | 0,84 |
| Altretamine | 1,18 | 1,03 | 0,88 |
| Carmofur | 0,39 | 0,39 | 0,99 |
| Mercaptopurine (6-MP) | 0,60 | 0,51 | 0,86 |
| Felbamate | 1,03 | 1,14 | 1,11 |
| Ivermectin | 0,91 | 0,99 | 1,09 |
| Clofarabine | 0,30 | 0,16 | 0,55 |
| Amonafide | 0,10 | 0,21 | 2,09 |
| Lopinavir | 0,89 | 1,03 | 1,17 |
| Flavopiridol (Alvocidib) | 0,18 | 0,23 | 1,28 |
| Leflunomide | 0,93 | 0,87 | 0,94 |
| Ramelteon | 1,03 | 1,01 | 0,98 |
| Amisulpride | 1,03 | 1,11 | 1,08 |
| Cetirizine DiHCl | 1,00 | 1,07 | 1,07 |
| Streptozotocin (STZ) | 0,88 | 1,20 | 1,36 |
| Fluconazole | 0,85 | 1,08 | 1,28 |
| Ketoconazole | 0,78 | 0,93 | 1,20 |
| Dacarbazine | 1,15 | 1,25 | 1,08 |
| Acitretin | 0,92 | 1,07 | 1,16 |
| Meropenem | 0,93 | 0,95 | 1,02 |
| Topotecan HCl | 0,12 | 0,12 | 0,95 |
| Enzalutamide (MDV3100) | 1,09 | 0,98 | 0,90 |
| Cinacalcet HCl | 1,09 | 1,00 | 0,92 |
| Aniracetam | 1,09 | 1,00 | 0,92 |
| Cilnidipine | 0,96 | 1,01 | 1,05 |
| Dexamethasone (DHAP) | 0,98 | 1,08 | 1,10 |
| Flumazenil | 0,99 | 1,10 | 1,11 |
| Lansoprazole | 0,90 | 1,04 | 1,15 |
| Dexrazoxane HCl (ICRF-187, ADR-529) | 1,27 | 1,04 | 0,82 |
| Bafetinib (INNO-406) | 0,79 | 0,77 | 0,98 |
| Mianserin HCl | 0,89 | 1,09 | 1,22 |
| Letrozole | 1,21 | 1,14 | 0,94 |
| Dienogest | 1,11 | 0,98 | 0,87 |
| Celecoxib | 0,96 | 0,86 | 0,90 |
| Artemisinin | 1,00 | 1,00 | 1,00 |
| Cilostazol | 0,94 | 0,96 | 1,03 |
| Doxazosin Mesylate | 0,94 | 1,06 | 1,13 |
| Fluoxetine HCl | 0,91 | 1,02 | 1,13 |
| Levetiracetam | 0,82 | 0,97 | 1,18 |
| Epirubicin HCl | 0,19 | 0,19 | 0,96 |
| Doripenem Hydrate | 0,91 | 1,04 | 1,15 |
| Mosapride Citrate | 0,87 | 0,90 | 1,04 |
| Temozolomide | 1,14 | 1,16 | 1,02 |
| Entecavir Hydrate | 1,29 | 0,95 | 0,73 |
| Avagacestat (BMS-708163) | 1,09 | 0,92 | 0,85 |
| Asenapine | 1,01 | 0,92 | 0,92 |
| Floxuridine | 0,27 | 0,11 | 0,42 |
| Edaravone | 1,01 | 1,26 | 1,25 |
| Fluvoxamine maleate | 1,00 | 0,87 | 0,86 |
| Lidocaine | 0,98 | 1,08 | 1,10 |
| Oxaliplatin | 0,35 | 0,38 | 1,09 |
| Gestodene | 0,99 | 1,00 | 1,02 |
| Nafamostat Mesylate | 0,97 | 0,96 | 0,99 |
| Tamoxifen | 1,18 | 1,09 | 0,93 |
| Nepafenac | 1,30 | 1,14 | 0,88 |
| Vemurafenib (PLX4032, RG7204) | 1,01 | 0,87 | 0,86 |
| Benazepril HCl | 1,04 | 0,98 | 0,95 |
| FT-207 (NSC 148958) | 0,99 | 1,02 | 1,02 |
| Ellagic acid | 0,91 | 1,08 | 1,19 |
| Gatifloxacin | 1,06 | 0,93 | 0,88 |
| Loratadine | 0,94 | 0,93 | 0,99 |
| Etoposide | 0,41 | 0,30 | 0,74 |
| Drospirenone | 0,89 | 1,00 | 1,12 |
| Omeprazole | 1,04 | 1,15 | 1,11 |
| Vincristine | 0,14 | 0,24 | 1,67 |
| Rufinamide | 1,21 | 1,18 | 0,97 |
| Acarbose | 1,09 | 1,08 | 1,00 |
| Budesonide | 1,05 | 1,08 | 1,03 |
| Ifosfamide | 0,96 | 1,00 | 1,04 |
| Etodolac | 1,14 | 1,27 | 1,11 |
| Genistein | 0,96 | 0,99 | 1,03 |
| Losartan Potassium (DuP 753) | 0,97 | 1,15 | 1,19 |
| Ondansetron HCl | 1,15 | 1,22 | 1,06 |
| VX-809 (Lumacaftor) | 1,27 | 1,12 | 0,88 |
| Semagacestat (LY450139) | 1,44 | 1,26 | 0,88 |
| Tenofovir | 1,19 | 1,30 | 1,09 |
| Ranolazine 2HCl | 1,16 | 1,15 | 0,99 |
| Topiramate | 1,17 | 1,33 | 1,13 |
| Cilomilast | 1,57 | 1,25 | 0,79 |
| TSU-68 (SU6668, Orantinib) | 1,40 | 1,31 | 0,94 |
| Ponatinib (AP24534) | 0,57 | 0,36 | 0,64 |
| Alprostadil | 1,34 | 1,38 | 1,03 |
| Quizartinib (AC220) | 0,59 | 0,68 | 1,15 |
| Oxcarbazepine | 1,07 | 0,97 | 0,90 |
| Pomalidomide | 1,31 | 1,17 | 0,89 |
| Reserpine | 1,30 | 1,02 | 0,79 |
| Tigecycline | 1,08 | 1,01 | 0,93 |
| Repaglinide | 1,17 | 0,95 | 0,81 |
| Tranilast | 1,06 | 0,94 | 0,89 |
| Zibotentan (ZD4054) | 1,30 | 1,06 | 0,81 |
| Safinamide Mesylate | 1,20 | 1,18 | 0,98 |
| Fludarabine | 1,27 | 0,96 | 0,75 |
| Norfloxacin | 1,22 | 1,14 | 0,93 |
| Telaprevir (VX-950) | 1,09 | 1,12 | 1,03 |
| Pelitinib (EKB-569) | 0,20 | 0,26 | 1,28 |
| Tazarotene | 1,19 | 1,04 | 0,87 |
| Furosemide | 1,23 | 1,05 | 0,85 |
| Trilostane | 1,27 | 1,19 | 0,94 |
| Rolipram | 1,21 | 1,11 | 0,91 |
| Venlafaxine | 1,27 | 1,09 | 0,86 |
| Atazanavir Sulfate | 0,97 | 0,81 | 0,84 |
| Pimasertib (AS-703026) | 1,11 | 0,56 | 0,51 |
| Pralatrexate | 0,22 | 0,19 | 0,86 |
| Lactulose | 1,10 | 0,94 | 0,86 |
| Saxagliptin | 1,12 | 1,16 | 1,03 |
| Pizotifen Malate | 0,95 | 1,12 | 1,18 |
| Fasudil (HA-1077) HCl | 1,17 | 1,14 | 0,97 |
| Olmesartan Medoxomil | 1,17 | 0,97 | 0,82 |
| Vecuronium Bromide | 0,96 | 1,12 | 1,17 |
| Sildenafil Citrate | 0,95 | 1,05 | 1,11 |
| Voriconazole | 0,94 | 1,05 | 1,12 |
| Ofloxacin | 1,22 | 0,98 | 0,80 |
| VX-222 (VCH-222, Lomibuvir) | 1,01 | 1,02 | 1,01 |
| Betamethasone | 1,06 | 1,09 | 1,02 |
| Tadalafil | 1,14 | 1,04 | 0,92 |
| EX 527 (Selisistat) | 1,16 | 1,11 | 0,95 |
| Resveratrol | 0,62 | 0,91 | 1,47 |
| BIRB 796 (Doramapimod) | 0,94 | 0,87 | 0,92 |
| Cefdinir | 1,17 | 1,05 | 0,90 |
| Linezolid | 1,08 | 1,20 | 1,11 |
| Sumatriptan Succinate | 0,79 | 0,98 | 1,25 |
| Zileuton | 0,90 | 0,95 | 1,05 |
| Marbofloxacin | 1,02 | 1,00 | 0,97 |
| Zosuquidar (LY335979) 3HCl | 0,85 | 0,90 | 1,05 |
| Mycophenolate Mofetil | 0,24 | 0,21 | 0,87 |
| Cyclosporine | 0,79 | 0,82 | 1,04 |
| Febuxostat | 1,05 | 1,14 | 1,08 |
| Rocuronium Bromide | 0,94 | 1,02 | 1,08 |
| Sulfasalazine | 1,01 | 1,01 | 1,00 |
| Clotrimazole | 1,27 | 1,06 | 0,83 |
| Alfuzosin HCl | 0,96 | 0,99 | 1,02 |
| Tamsulosin | 1,00 | 1,07 | 1,07 |
| Ziprasidone HCl | 1,04 | 1,02 | 0,98 |
| Moxifloxacin HCl | 1,14 | 1,04 | 0,91 |
| Daclatasvir (BMS-790052) | 0,97 | 1,05 | 1,07 |
| Dyphylline | 1,11 | 0,98 | 0,89 |
| Pracinostat (SB939) | 0,07 | 0,06 | 0,95 |
| Dapagliflozin | 1,20 | 1,42 | 1,18 |
| Stavudine (d4T) | 1,05 | 1,05 | 1,00 |
| Candesartan | 1,12 | 1,03 | 0,93 |
| Rizatriptan Benzoate | 1,15 | 1,19 | 1,04 |
| Clopidogrel | 0,97 | 1,08 | 1,11 |
| Tianeptine sodium | 1,03 | 1,07 | 1,04 |
| Zonisamide | 1,03 | 1,09 | 1,05 |
| Doxercalciferol | 1,19 | 1,04 | 0,87 |
| Iloperidone | 0,99 | 0,99 | 1,00 |
| Aztreonam | 1,06 | 0,95 | 0,90 |
| Natamycin | 1,07 | 1,02 | 0,95 |
| Nebivolol | 0,99 | 1,08 | 1,09 |
| Tenofovir Disoproxil Fumarate | 1,04 | 1,08 | 1,04 |
| Apixaban | 1,30 | 1,07 | 0,82 |
| Pyridostigmine Bromide | 1,23 | 1,20 | 0,98 |
| Prazosin HCl | 0,95 | 1,25 | 1,31 |
| Tizanidine HCl | 1,08 | 1,15 | 1,06 |
| Ispinesib (SB-715992) | 0,10 | 0,13 | 1,22 |
| Alfacalcidol | 0,17 | 0,14 | 0,83 |
| Naratriptan | 1,07 | 1,21 | 1,13 |
| Irbesartan | 1,08 | 1,18 | 1,09 |
| SAR245409 (XL765) | 1,02 | 1,18 | 1,16 |
| Pimobendan | 1,18 | 1,13 | 0,96 |
| Methimazole | 1,25 | 1,27 | 1,01 |
| Hydrochlorothiazide | 1,14 | 1,18 | 1,04 |
| Adefovir Dipivoxil | 0,37 | 0,23 | 0,64 |
| Prilocaine | 1,12 | 1,12 | 1,00 |
| Nitazoxanide | 0,95 | 0,95 | 1,00 |
| Ibuprofen | 1,07 | 1,12 | 1,05 |
| Ketorolac | 0,95 | 1,19 | 1,25 |
| Enalaprilat Dihydrate | 0,98 | 0,92 | 0,94 |
| Aminoglutethimide | 1,21 | 0,93 | 0,77 |
| Sulfanilamide | 1,23 | 1,10 | 0,90 |
| Torsemide | 1,21 | 0,89 | 0,74 |
| Metolazone | 1,01 | 1,02 | 1,00 |
| Estradiol | 1,07 | 0,84 | 0,78 |
| Zalcitabine | 1,19 | 0,94 | 0,79 |
| Darunavir Ethanolate | 0,99 | 0,97 | 0,98 |
| Triamcinolone Acetonide | 1,01 | 0,91 | 0,90 |
| Amprenavir | 0,99 | 0,96 | 0,97 |
| Adenosine | 1,00 | 0,99 | 0,99 |
| Dofetilide | 1,08 | 0,81 | 0,76 |
| Aminophylline | 1,11 | 0,86 | 0,77 |
| Betamethasone Dipropionate | 1,31 | 0,88 | 0,67 |
| Desonide | 1,15 | 0,81 | 0,71 |
| Cefoperazone | 1,02 | 1,05 | 1,03 |
| Deferasirox | 0,73 | 0,61 | 0,84 |
| Azathioprine | 1,10 | 0,82 | 0,75 |
| Theophylline | 0,93 | 0,93 | 1,00 |
| Orlistat | 1,04 | 0,94 | 0,91 |
| Albendazole | 0,09 | 0,13 | 1,50 |
| Zolmitriptan | 0,96 | 0,97 | 1,02 |
| Isradipine | 1,03 | 0,67 | 0,65 |
| Amorolfine HCl | 1,08 | 0,92 | 0,86 |
| Meprednisone | 1,14 | 1,00 | 0,88 |
| Didanosine | 1,03 | 0,96 | 0,93 |
| Silodosin | 0,98 | 0,97 | 0,99 |
| Piroxicam | 1,02 | 1,21 | 1,19 |
| Indomethacin | 1,35 | 1,11 | 0,83 |
| Prednisone | 0,91 | 0,99 | 1,09 |
| Allopurinol | 1,06 | 0,94 | 0,89 |
| Chlorothiazide | 0,95 | 0,92 | 0,97 |
| Telbivudine | 1,01 | 1,14 | 1,13 |
| Estrone | 0,96 | 0,94 | 0,98 |
| Chloramphenicol | 1,01 | 0,89 | 0,88 |
| Betamethasone valerate (Betnovate) | 1,02 | 0,96 | 0,93 |
| Divalproex Sodium | 1,06 | 0,95 | 0,89 |
| Riluzole | 0,87 | 0,97 | 1,12 |
| Gemcitabine | 0,26 | 0,17 | 0,65 |
| Paliperidone | 1,21 | 1,03 | 0,85 |
| Acetylcysteine | 0,89 | 1,01 | 1,13 |
| Zafirlukast | 0,91 | 0,85 | 0,94 |
| Methyldopa | 1,00 | 0,88 | 0,88 |
| Monobenzone | 0,69 | 0,83 | 1,20 |
| Flucytosine | 0,91 | 0,99 | 1,08 |
| Flurbiprofen | 0,98 | 0,94 | 0,95 |
| Praziquantel | 0,94 | 0,98 | 1,04 |
| Emtricitabine | 1,03 | 1,14 | 1,11 |
| Risperidone | 1,08 | 0,92 | 0,85 |
| Glipizide | 1,00 | 1,02 | 1,01 |
| Terbinafine | 1,07 | 1,17 | 1,09 |
| Alendronate | 0,96 | 1,02 | 1,06 |
| Acetaminophen | 0,98 | 1,02 | 1,04 |
| Ursodiol | 1,08 | 0,98 | 0,91 |
| Tretinoin | 1,08 | 1,13 | 1,04 |
| Trichlormethiazide | 0,94 | 1,04 | 1,10 |
| Disulfiram | 0,17 | 0,03 | 0,17 |
| Busulfan | 0,97 | 0,88 | 0,91 |
| Progesterone | 0,95 | 0,89 | 0,93 |
| Sulfapyridine | 0,88 | 0,95 | 1,07 |
| Glyburide | 1,17 | 0,86 | 0,73 |
| Levonorgestrel | 1,22 | 1,04 | 0,85 |
| Ethinyl Estradiol | 0,96 | 0,95 | 0,98 |
| Erythromycin | 0,99 | 0,76 | 0,77 |
| Nitrofural | 0,92 | 0,99 | 1,09 |
| Phenylbutazone | 0,93 | 1,11 | 1,20 |
| Loteprednol etabonate | 0,99 | 0,82 | 0,83 |
| Mesalamine | 0,96 | 0,79 | 0,82 |
| Carbamazepine | 1,02 | 0,69 | 0,68 |
| Lamivudine | 1,18 | 0,90 | 0,76 |
| Sulfameter | 0,96 | 1,09 | 1,13 |
| Fomepizole | 1,09 | 1,21 | 1,12 |
| Gemfibrozil | 1,04 | 1,16 | 1,12 |
| Naproxen | 0,97 | 1,15 | 1,19 |
| Amphotericin B | 1,01 | 1,10 | 1,09 |
| Ketoprofen | 0,86 | 1,15 | 1,33 |
| Ezetimibe | 0,91 | 1,14 | 1,25 |
| （6-）ε-​Aminocaproic acid | 1,13 | 1,19 | 1,06 |
| Ipratropium Bromide | 0,91 | 1,22 | 1,33 |
| Hydrocortisone | 0,90 | 1,35 | 1,49 |
| Eplerenone | 0,99 | 1,49 | 1,51 |
| Indapamide | 1,18 | 1,36 | 1,15 |
| Diltiazem HCl | 1,00 | 1,23 | 1,23 |
| Sparfloxacin | 0,93 | 1,17 | 1,26 |
| Thiabendazole | 1,20 | 1,19 | 0,99 |
| Oxybutynin | 1,39 | 1,52 | 1,09 |
| Cefditoren Pivoxil | 1,16 | 1,35 | 1,17 |
| Vidarabine | 1,17 | 1,23 | 1,06 |
| Ranolazine | 1,17 | 1,20 | 1,03 |
| Amlodipine besylate (Norvasc) | 0,97 | 1,02 | 1,06 |
| Carvedilol | 0,92 | 1,07 | 1,16 |
| Chenodeoxycholic Acid | 1,00 | 1,29 | 1,30 |
| Mitotane | 1,31 | 1,24 | 0,94 |
| Diphenhydramine HCl | 1,08 | 1,21 | 1,12 |
| Felodipine | 0,91 | 0,92 | 1,01 |
| Guaifenesin | 1,36 | 1,24 | 0,91 |
| Enoxacin | 1,24 | 1,26 | 1,02 |
| Sulfadiazine | 1,04 | 1,07 | 1,03 |
| Teniposide | 0,17 | 0,14 | 0,82 |
| Ranitidine | 1,14 | 1,04 | 0,91 |
| Chlorpheniramine Maleate | 1,24 | 1,13 | 0,91 |
| Atracurium Besylate | 1,29 | 1,44 | 1,11 |
| Cimetidine | 1,01 | 1,06 | 1,05 |
| Methylprednisolone | 1,20 | 1,22 | 1,02 |
| Dapoxetine HCl | 0,99 | 1,06 | 1,07 |
| Deflazacort | 1,12 | 0,96 | 0,85 |
| Rifabutin | 1,17 | 1,19 | 1,01 |
| Pitavastatin Calcium | 0,44 | 0,12 | 0,28 |
| Chlorprothixene | 1,06 | 1,05 | 1,00 |
| Stanozolol | 1,11 | 1,26 | 1,14 |
| Acadesine | 1,07 | 1,14 | 1,06 |
| Fenoprofen Calcium | 1,06 | 1,18 | 1,11 |
| Butoconazole nitrate | 1,05 | 1,24 | 1,18 |
| Clemastine Fumarate | 1,16 | 1,14 | 0,98 |
| Meloxicam | 1,15 | 1,14 | 1,00 |
| Valaciclovir HCl | 1,03 | 1,12 | 1,10 |
| Nizatidine | 1,11 | 1,07 | 0,97 |
| Nevirapine | 1,19 | 1,12 | 0,94 |
| Rifapentine | 1,24 | 1,01 | 0,82 |
| Oxytetracycline (Terramycin) | 1,07 | 1,19 | 1,12 |
| Tetrabenazine (Xenazine) | 1,09 | 1,16 | 1,07 |
| Acetylcholine Chloride | 1,10 | 1,09 | 0,99 |
| Erdosteine | 1,11 | 1,19 | 1,07 |
| Azithromycin | 1,05 | 1,05 | 1,00 |
| Curcumin | 1,06 | 1,30 | 1,22 |
| Mesna | 1,28 | 1,21 | 0,94 |
| Ganciclovir | 1,07 | 0,99 | 0,92 |
| Carbidopa | 0,92 | 0,98 | 1,06 |
| Esomeprazole Magnesium | 1,17 | 1,16 | 0,99 |
| Suprofen | 1,33 | 1,07 | 0,80 |
| Toremifene Citrate | 1,00 | 1,12 | 1,13 |
| Rifaximin | 1,06 | 1,10 | 1,04 |
| Acipimox | 1,06 | 1,13 | 1,06 |
| Betaxolol hydrochloride (Betoptic) | 0,96 | 1,09 | 1,13 |
| Albendazole Oxide | 1,05 | 0,92 | 0,87 |
| Bifonazole | 0,94 | 0,96 | 1,02 |
| Methocarbamol | 1,31 | 1,17 | 0,89 |
| Roxatidine Acetate HCl | 0,93 | 0,89 | 0,96 |
| Valsartan | 0,97 | 0,92 | 0,95 |
| Nicotinic Acid | 1,24 | 1,29 | 1,04 |
| Pyrazinamide | 1,32 | 1,33 | 1,00 |
| Ethionamide | 0,95 | 1,16 | 1,22 |
| Simvastatin | 0,50 | 0,67 | 1,33 |
| Aciclovir | 1,02 | 0,93 | 0,92 |
| Proparacaine HCl | 1,01 | 0,99 | 0,98 |
| Flubendazole | 0,29 | 0,22 | 0,78 |
| Pefloxacin Mesylate | 0,92 | 1,00 | 1,08 |
| Prednisolone | 1,32 | 1,24 | 0,94 |
| Protionamide | 1,09 | 1,13 | 1,04 |
| Dipyridamole | 1,04 | 0,94 | 0,91 |
| Nimodipine | 1,07 | 1,36 | 1,27 |
| Quetiapine Fumarate | 1,29 | 1,13 | 0,88 |
| Trifluridine | 0,40 | 0,30 | 0,75 |
| Ramipril | 1,06 | 1,16 | 1,09 |
| Nifedipine | 1,00 | 0,99 | 0,99 |
| Pranlukast | 1,09 | 1,16 | 1,06 |
| Chloroxine | 0,76 | 0,29 | 0,38 |
| Metoprolol Tartrate | 1,03 | 1,12 | 1,09 |
| Telmisartan | 1,19 | 1,08 | 0,90 |
| Idoxuridine | 1,05 | 0,96 | 0,91 |
| Hydroxyurea | 1,12 | 1,18 | 1,05 |
| Nisoldipine | 0,90 | 0,80 | 0,88 |
| Rifampin | 1,46 | 1,24 | 0,85 |
| Azacitidine | 0,54 | 0,65 | 1,20 |
| Fenofibrate | 1,30 | 1,14 | 0,88 |
| Amiloride HCl | 1,12 | 1,19 | 1,07 |
| Oxfendazole | 1,14 | 1,17 | 1,03 |
| Lomustine | 1,29 | 1,25 | 0,97 |
| Diethylstilbestrol | 1,16 | 1,14 | 0,98 |
| Potassium Iodide | 1,33 | 1,43 | 1,07 |
| Memantine HCl | 1,06 | 1,20 | 1,13 |
| Gimeracil | 1,01 | 1,24 | 1,23 |
| Flutamide | 1,35 | 1,35 | 1,00 |
| Haloperidol | 1,28 | 1,23 | 0,96 |
| Enalapril Maleate | 1,03 | 1,33 | 1,29 |
| Sulphadimethoxine | 0,91 | 1,20 | 1,32 |
| Sarafloxacin HCl | 1,08 | 1,22 | 1,12 |
| Elvitegravir (GS-9137, JTK-303) | 0,82 | 0,71 | 0,87 |
| Formoterol Hemifumarate | 0,97 | 1,23 | 1,26 |
| Rebamipide | 1,16 | 1,22 | 1,05 |
| Tropisetron | 1,26 | 1,41 | 1,12 |
| Cyproheptadine HCl | 0,94 | 1,03 | 1,09 |
| Cyclophosphamide Monohydrate | 0,89 | 1,08 | 1,22 |
| Fluvastatin Sodium | 0,30 | 0,32 | 1,09 |
| Phenindione | 0,95 | 1,13 | 1,19 |
| Menadione | 0,99 | 1,02 | 1,04 |
| Rimantadine | 0,94 | 0,97 | 1,03 |
| Methscopolamine | 1,03 | 1,12 | 1,08 |
| Maraviroc | 1,20 | 1,21 | 1,01 |
| Chlormezanone | 0,93 | 1,17 | 1,26 |
| Epalrestat | 1,02 | 1,04 | 1,02 |
| Nicotinamide (Vitamin B3) | 1,11 | 1,23 | 1,11 |
| Doxifluridine | 0,99 | 0,90 | 0,91 |
| Tolnaftate | 0,90 | 0,95 | 1,06 |
| Tioconazole | 1,25 | 1,15 | 0,91 |
| Alibendol | 1,01 | 1,08 | 1,07 |
| Methoxsalen | 1,06 | 0,97 | 0,92 |
| Primidone | 1,10 | 1,02 | 0,93 |
| Amiodarone HCl | 1,09 | 0,93 | 0,86 |
| Raltegravir (MK-0518) | 0,99 | 0,96 | 0,97 |
| Ketotifen Fumarate | 0,90 | 0,98 | 1,09 |
| Aspartame | 1,12 | 0,91 | 0,81 |
| Vitamin B12 | 1,16 | 1,23 | 1,05 |
| Pioglitazone HCl | 0,90 | 0,90 | 0,99 |
| Terazosin HCl | 0,87 | 0,91 | 1,04 |
| Tropicamide | 1,15 | 1,22 | 1,06 |
| Irsogladine | 1,08 | 1,01 | 0,93 |
| Miconazole Nitrate | 1,06 | 1,06 | 1,00 |
| Nefiracetam | 1,01 | 1,04 | 1,02 |
| Meclizine 2HCl | 1,00 | 1,08 | 1,08 |
| Pyrimethamine | 0,59 | 1,06 | 1,79 |
| Urapidil HCl | 0,98 | 1,06 | 1,08 |
| Candesartan Cilexetil | 1,21 | 0,94 | 0,78 |
| Diclofenac Sodium | 1,10 | 1,20 | 1,10 |
| Lornoxicam | 0,89 | 0,96 | 1,08 |
| Bromhexine HCl | 0,93 | 1,07 | 1,15 |
| Pregnenolone | 1,22 | 1,19 | 0,98 |
| Triamcinolone | 1,09 | 1,09 | 1,00 |
| Sulfamethizole | 1,09 | 1,14 | 1,05 |
| Nicorandil | 1,04 | 0,98 | 0,95 |
| Mometasone furoate | 1,14 | 1,00 | 0,87 |
| Sulindac | 1,00 | 1,01 | 1,01 |
| Diclazuril | 0,98 | 0,92 | 0,94 |
| Phentolamine Mesylate | 1,16 | 0,96 | 0,83 |
| Avobenzone | 1,32 | 1,22 | 0,93 |
| Captopril | 0,95 | 1,01 | 1,06 |
| Lovastatin | 0,43 | 0,55 | 1,27 |
| Sulfamethoxazole | 1,15 | 1,11 | 0,96 |
| Nystatin (Fungicidin) | 1,07 | 0,93 | 0,87 |
| Sulbactam | 1,15 | 0,94 | 0,82 |
| Tamoxifen Citrate | 1,05 | 0,88 | 0,84 |
| Propylthiouracil | 1,04 | 0,94 | 0,91 |
| Pramipexole dihydrochloride monohydrate | 1,00 | 0,96 | 0,96 |
| Uridine | 0,91 | 1,05 | 1,15 |
| Nimesulide | 1,12 | 1,10 | 0,98 |
| Amlodipine | 1,13 | 1,17 | 1,04 |
| Oxytetracycline Dihydrate | 1,01 | 1,19 | 1,18 |
| Tiopronin | 0,91 | 1,09 | 1,20 |
| Sulfisoxazole | 1,25 | 1,17 | 0,93 |
| Isoniazid | 1,08 | 1,13 | 1,05 |
| Tolfenamic Acid | 1,25 | 1,05 | 0,84 |
| Meglumine | 1,05 | 1,05 | 1,00 |
| Fluticasone propionate | 1,12 | 1,13 | 1,01 |
| Mirtazapine | 0,99 | 1,28 | 1,29 |
| Flunarizine 2HCl | 1,05 | 1,22 | 1,16 |
| Dyclonine HCl | 1,44 | 1,41 | 0,98 |
| Metronidazole | 1,36 | 1,34 | 0,99 |
| Orphenadrine Citrate | 1,32 | 1,26 | 0,95 |
| Balofloxacin | 1,35 | 1,19 | 0,88 |
| Crystal Violet | 0,14 | 0,21 | 1,42 |
| Levofloxacin | 1,25 | 1,29 | 1,03 |
| Pranoprofen | 1,43 | 1,08 | 0,75 |
| Aripiprazole | 1,23 | 1,12 | 0,91 |
| Lacidipine | 1,19 | 1,21 | 1,01 |
| Benidipine HCl | 1,21 | 1,04 | 0,86 |
| Fenticonazole Nitrate | 1,38 | 1,10 | 0,80 |
| Cyproterone Acetate | 1,60 | 1,26 | 0,79 |
| Lafutidine | 1,07 | 1,38 | 1,29 |
| Nalidixic acid | 1,26 | 1,06 | 0,84 |
| Tolbutamide | 1,23 | 1,28 | 1,04 |
| Moexipril HCl | 1,14 | 1,17 | 1,03 |
| Dexmedetomidine HCl (Precedex) | 1,04 | 1,32 | 1,27 |
| Rasagiline Mesylate | 1,10 | 1,40 | 1,28 |
| Dronedarone HCl | 1,14 | 1,18 | 1,03 |
| Naftopidil | 1,03 | 1,07 | 1,04 |
| Dabigatran Etexilate | 1,09 | 1,23 | 1,13 |
| Aliskiren Hemifumarate | 1,09 | 1,25 | 1,15 |
| LY2157299 | 1,05 | 1,22 | 1,16 |
| Moxonidine | 1,04 | 1,21 | 1,16 |
| Orotic acid (6-Carboxyuracil) | 1,06 | 1,03 | 0,96 |
| Levosimendan | 0,81 | 0,80 | 0,98 |
| Clevidipine Butyrate | 0,92 | 0,90 | 0,98 |
| Betaxolol | 1,09 | 1,24 | 1,14 |
| Naltrexone HCl | 0,92 | 1,16 | 1,26 |
| Conivaptan HCl | 0,95 | 1,10 | 1,16 |
| S- (+)-Rolipram | 1,12 | 1,22 | 1,09 |
| Taladegib (LY2940680) | 1,14 | 1,10 | 0,97 |
| R788 (Fostamatinib) Disodium | 0,47 | 0,22 | 0,47 |
| Esomeprazole Sodium | 1,10 | 1,12 | 1,01 |
| Ozagrel HCl | 1,18 | 1,18 | 1,00 |
| Piperine | 1,01 | 1,11 | 1,10 |
| Amantadine HCl | 1,17 | 1,28 | 1,09 |
| Cilazapril Monohydrate | 0,95 | 1,05 | 1,11 |
| Detomidine HCl | 0,91 | 1,17 | 1,30 |
| Levosulpiride | 0,96 | 1,06 | 1,11 |
| Ibutilide Fumarate | 1,05 | 1,14 | 1,08 |
| Bazedoxifene HCl | 0,96 | 1,15 | 1,20 |
| Tebipenem Pivoxil | 1,01 | 1,21 | 1,19 |
| Formestane | 1,02 | 1,23 | 1,20 |
| Volasertib (BI 6727) | 0,07 | 0,08 | 1,20 |
| Argatroban | 1,07 | 1,20 | 1,12 |
| Rutin | 0,97 | 1,06 | 1,09 |
| Amfebutamone HCl | 1,22 | 1,31 | 1,07 |
| Adiphenine HCl | 0,92 | 1,12 | 1,22 |
| Almotriptan Malate | 1,06 | 1,17 | 1,10 |
| Flunixin Meglumin | 1,03 | 1,06 | 1,03 |
| Probucol | 0,91 | 1,16 | 1,28 |
| Atropine | 0,89 | 1,14 | 1,27 |
| Bazedoxifene Acetate | 1,00 | 1,08 | 1,08 |
| Mubritinib (TAK 165) | 0,60 | 0,62 | 1,02 |
| Fesoterodine Fumarate | 1,17 | 1,02 | 0,87 |
| Mecarbinate | 0,88 | 0,83 | 0,94 |
| Silibinin | 0,92 | 1,08 | 1,18 |
| Benserazide HCl | 1,06 | 1,22 | 1,15 |
| Duloxetine HCl | 0,96 | 0,94 | 0,97 |
| Ambrisentan | 1,05 | 1,02 | 0,97 |
| Imidapril HCl | 1,00 | 0,93 | 0,93 |
| Licofelone | 0,93 | 1,04 | 1,11 |
| Roflumilast | 0,90 | 1,14 | 1,27 |
| Rosuvastatin Calcium | 1,11 | 1,02 | 0,92 |
| Irinotecan HCl Trihydrate | 0,40 | 0,38 | 0,95 |
| CX-4945 (Silmitasertib) | 0,93 | 0,66 | 0,72 |
| Rosiglitazone HCl | 1,09 | 0,96 | 0,88 |
| D-Mannitol | 1,03 | 1,11 | 1,08 |
| Bupivacaine HCl | 1,50 | 1,30 | 0,87 |
| Trimebutine | 0,98 | 0,88 | 0,90 |
| Bexarotene | 0,99 | 1,05 | 1,06 |
| Vinpocetine | 0,97 | 1,02 | 1,05 |
| Dextrose | 1,06 | 1,01 | 0,96 |
| Neratinib (HKI-272) | 0,39 | 0,57 | 1,47 |
| Telotristat Etiprate (LX 1606 Hippurate) | 0,84 | 0,78 | 0,93 |
| Apatinib | 0,77 | 0,72 | 0,93 |
| Artemether | 0,70 | 0,84 | 1,20 |
| Atorvastatin Calcium | 0,56 | 0,57 | 1,03 |
| L-carnitine | 1,17 | 1,23 | 1,06 |
| Bethanechol chloride | 1,32 | 1,26 | 0,95 |
| Ivabradine HCl | 1,10 | 1,05 | 0,95 |
| Temocapril HCl | 1,11 | 1,08 | 0,97 |
| Lapatinib | 0,95 | 0,92 | 0,96 |
| Xylose | 1,02 | 1,05 | 1,03 |
| LDE225 (NVP-LDE225,Erismodegib) | 1,04 | 1,04 | 1,00 |
| LY2784544 | 0,44 | 0,46 | 1,05 |
| CAL-101 (Idelalisib, GS-1101) | 1,05 | 0,94 | 0,89 |
| Cyclosporin A | 0,89 | 0,98 | 1,09 |
| Famotidine | 1,41 | 1,15 | 0,81 |
| Sorbitol | 1,30 | 1,25 | 0,96 |
| Chlorpromazine HCl | 1,74 | 1,64 | 0,94 |
| Rivastigmine Tartrate | 1,25 | 1,17 | 0,93 |
| Gabexate Mesylate | 1,28 | 1,26 | 0,99 |
| Cisatracurium Besylate | 1,38 | 1,36 | 0,98 |
| Mestranol | 1,25 | 1,12 | 0,89 |
| Sitafloxacin Hydrate | 1,22 | 1,20 | 0,98 |
| MLN2238 | 0,03 | 0,04 | 1,08 |
| Eltrombopag Olamine | 0,96 | 0,90 | 0,94 |
| DL-Carnitine HCl | 1,19 | 1,11 | 0,93 |
| Clindamycin HCl | 0,97 | 1,18 | 1,22 |
| Medroxyprogesterone acetate | 0,91 | 0,93 | 1,02 |
| Quinapril HCl | 0,85 | 1,15 | 1,35 |
| Gallamine Triethiodide | 0,87 | 1,14 | 1,31 |
| Mitoxantrone HCl | 0,03 | 0,09 | 3,23 |
| Oxymetazoline hydrochloride | 1,02 | 1,25 | 1,23 |
| Roxithromycin | 0,96 | 1,10 | 1,14 |
| Maprotiline HCl | 0,90 | 0,92 | 1,03 |
| Ritodrine HCl | 1,07 | 1,06 | 0,99 |
| Ceftiofur HCl | 0,88 | 0,80 | 0,90 |
| Rosiglitazone | 0,96 | 1,08 | 1,13 |
| Clonidine HCl | 0,85 | 1,03 | 1,22 |
| Phenylephrine HCl | 0,72 | 0,93 | 1,30 |
| Trazodone HCl | 0,91 | 0,98 | 1,08 |
| Imatinib (STI571) | 0,88 | 1,01 | 1,14 |
| Moroxydine HCl | 0,77 | 1,05 | 1,36 |
| Ozagrel | 0,98 | 0,85 | 0,87 |
| Scopolamine HBr | 0,81 | 0,98 | 1,22 |
| Naphazoline HCl | 0,78 | 1,01 | 1,30 |
| Isoconazole nitrate | 1,00 | 0,99 | 0,99 |
| Tiotropium Bromide hydrate | 0,87 | 0,94 | 1,08 |
| Terbinafine HCl | 0,94 | 1,03 | 1,09 |
| Clozapine | 0,87 | 0,93 | 1,07 |
| Prednisolone Acetate | 0,94 | 0,95 | 1,01 |
| Thiamphenicol | 1,37 | 1,24 | 0,91 |
| Itraconazole | 0,85 | 0,94 | 1,12 |
| Mycophenolic acid | 0,16 | 0,21 | 1,34 |
| Pancuronium dibromide | 0,81 | 0,86 | 1,06 |
| Sotalol | 0,91 | 1,02 | 1,13 |
| Epinephrine Bitartrate | 0,77 | 0,85 | 1,11 |
| Econazole nitrate | 1,09 | 0,81 | 0,74 |
| Trospium chloride | 0,91 | 0,91 | 0,99 |
| Cortisone acetate | 1,03 | 0,87 | 0,84 |
| Pramipexole | 0,78 | 0,93 | 1,19 |
| Tetracaine HCl | 1,13 | 1,06 | 0,94 |
| Clobetasol propionate | 1,06 | 0,89 | 0,84 |
| Lincomycin HCl | 0,85 | 0,86 | 1,01 |
| Nateglinide | 0,79 | 0,94 | 1,18 |
| Phenoxybenzamine HCl | 0,75 | 0,82 | 1,10 |
| Spectinomycin HCl | 0,85 | 0,87 | 1,02 |
| L-Adrenaline | 0,86 | 0,79 | 0,92 |
| Miconazole | 1,08 | 0,81 | 0,75 |
| Tolterodine tartrate | 0,97 | 0,81 | 0,84 |
| Amiloride hydrochloride dihydrate | 1,06 | 0,93 | 0,87 |
| Domperidone | 0,92 | 0,81 | 0,88 |
| Tetracycline HCl | 1,28 | 1,17 | 0,92 |
| Brompheniramine hydrogen maleate | 1,03 | 0,99 | 0,96 |
| Loperamide HCl | 0,93 | 0,86 | 0,93 |
| Nitrendipine | 0,82 | 0,93 | 1,14 |
| Propafenone HCl | 0,92 | 0,86 | 0,93 |
| Sulfadoxine | 0,84 | 0,96 | 1,14 |
| Phenytoin sodium | 1,00 | 0,94 | 0,94 |
| Secnidazole | 1,04 | 1,05 | 1,01 |
| Sulbactam sodium | 0,81 | 1,13 | 1,39 |
| Clomifene citrate | 0,97 | 0,69 | 0,71 |
| Estriol | 0,77 | 0,72 | 0,94 |
| Xylometazoline HCl | 1,11 | 1,11 | 1,00 |
| Dimethyl Fumarate | 1,09 | 1,13 | 1,04 |
| Manidipine | 0,79 | 0,78 | 0,99 |
| Novobiocin Sodium | 0,88 | 0,76 | 0,87 |
| Racecadotril | 0,85 | 0,84 | 1,00 |
| Tenoxicam | 0,81 | 0,76 | 0,94 |
| Phenytoin | 0,80 | 0,78 | 0,97 |
| Acetanilide | 1,13 | 0,84 | 0,74 |
| Azelastine HCl | 0,94 | 0,78 | 0,83 |
| Cloxacillin Sodium | 1,01 | 0,99 | 0,98 |
| Famciclovir | 0,82 | 1,04 | 1,26 |
| Phenacetin | 1,02 | 0,98 | 0,96 |
| Miglitol | 1,25 | 0,92 | 0,74 |
| Manidipine 2HCl | 0,90 | 1,14 | 1,27 |
| Olanzapine | 0,91 | 1,06 | 1,17 |
| Ribavirin | 0,91 | 1,10 | 1,21 |
| Vardenafil HCl Trihydrate | 0,91 | 1,06 | 1,17 |
| Ciclopirox | 0,32 | 0,50 | 1,57 |
| Clomipramine HCl | 1,51 | 1,21 | 0,80 |
| 5-Aminolevulinic acid HCl | 1,00 | 1,00 | 1,00 |
| Amoxicillin Sodium | 1,03 | 1,02 | 0,99 |
| Fluocinolone Acetonide | 1,24 | 1,09 | 0,87 |
| Zidovudine | 1,14 | 1,07 | 0,93 |
| Pioglitazone | 1,08 | 1,10 | 1,02 |
| Milrinone | 1,21 | 1,26 | 1,04 |
| Olopatadine HCl | 1,29 | 1,09 | 0,84 |
| Rosiglitazone maleate | 1,28 | 1,09 | 0,85 |
| Xylazine HCl | 1,25 | 1,06 | 0,85 |
| Dopamine HCl | 1,15 | 1,12 | 0,97 |
| Phenformin HCl | 1,36 | 1,03 | 0,76 |
| Clarithromycin | 1,09 | 1,18 | 1,08 |
| Isoprenaline HCl | 1,13 | 1,11 | 0,97 |
| Tolvaptan | 1,35 | 1,32 | 0,98 |
| Cinepazide maleate | 1,14 | 1,03 | 0,90 |
| Enrofloxacin | 1,13 | 1,07 | 0,95 |
| Idebenone | 1,25 | 1,15 | 0,92 |
| Fostamatinib (R788) | 0,57 | 0,36 | 0,63 |
| Dacomitinib (PF299804, PF299) | 0,65 | 0,51 | 0,79 |
| Dovitinib (TKI-258) Dilactic Acid | 0,29 | 0,54 | 1,82 |
| Tideglusib | 1,33 | 1,29 | 0,97 |
| Pirfenidone | 1,22 | 1,26 | 1,04 |
| Amoxicillin | 1,20 | 1,14 | 0,95 |
| Linagliptin | 1,01 | 1,06 | 1,05 |
| Pramiracetam | 1,22 | 1,23 | 1,01 |
| Azilsartan | 1,11 | 1,08 | 0,97 |
| Medetomidine HCl | 1,15 | 0,93 | 0,81 |
| Mifepristone | 1,23 | 1,25 | 1,02 |
| GSK2126458 (GSK458) | 0,14 | 0,17 | 1,23 |
| Crenolanib (CP-868596) | 0,59 | 0,52 | 0,89 |
| Tofacitinib (CP-690550,Tasocitinib) | 1,20 | 1,27 | 1,06 |
| Clindamycin | 1,27 | 1,18 | 0,93 |
| Evacetrapib (LY2484595) | 0,89 | 0,91 | 1,02 |
| Aspirin | 1,10 | 1,11 | 1,01 |
| Bindarit | 1,25 | 1,09 | 0,87 |
| Clindamycin palmitate HCl | 1,34 | 1,22 | 0,91 |
| Otilonium Bromide | 1,19 | 0,98 | 0,82 |
| Epinephrine HCl | 1,05 | 0,97 | 0,93 |
| Buflomedil HCl | 1,14 | 1,08 | 0,95 |
| Ciprofibrate | 1,14 | 1,11 | 0,98 |
| TG101348 (SAR302503) | 0,47 | 0,46 | 0,99 |
| Sotrastaurin | 1,16 | 1,04 | 0,90 |
| Carfilzomib (PR-171) | 0,01 | 0,00 | 0,37 |
| Carbazochrome sodium sulfonate (AC-17) | 1,33 | 1,24 | 0,93 |
| Niflumic acid | 1,08 | 1,06 | 0,99 |
| Vildagliptin (LAF-237) | 1,13 | 1,03 | 0,91 |
| Oseltamivir Phosphate | 1,30 | 1,17 | 0,90 |
| Bosentan Hydrate | 1,09 | 1,20 | 1,10 |
| Diclofenac Potassium | 0,99 | 0,86 | 0,87 |
| Fluocinonide | 1,20 | 1,22 | 1,01 |
| Dolutegravir (GSK1349572) | 1,73 | 1,65 | 0,96 |
| Tivantinib (ARQ 197) | 0,13 | 0,16 | 1,22 |
| Sofosbuvir (PSI-7977, GS-7977) | 1,11 | 1,16 | 1,05 |
| Alogliptin | 1,09 | 1,17 | 1,07 |
| Clevudine | 1,16 | 1,18 | 1,02 |
| Ciclopirox ethanolamine | 0,37 | 0,58 | 1,56 |
| Daunorubicin HCl | 0,11 | 0,13 | 1,15 |
| L-Thyroxine | 1,33 | 1,27 | 0,96 |
| Rupatadine Fumarate | 1,10 | 0,92 | 0,84 |
| Diclofenac Diethylamine | 1,02 | 0,92 | 0,90 |
| Lonidamine | 1,33 | 1,16 | 0,87 |
| Trametinib (GSK1120212) | 1,21 | 0,76 | 0,63 |
| Varlitinib | 1,09 | 1,03 | 0,94 |
| Lonafarnib | 0,92 | 1,05 | 1,14 |
| Camostat Mesilate | 1,10 | 1,08 | 0,98 |
| Rivaroxaban | 1,09 | 1,18 | 1,08 |
| Rimonabant | 1,14 | 1,07 | 0,94 |
| Pravastatin sodium | 1,00 | 1,06 | 1,07 |
| Gliclazide | 1,28 | 1,30 | 1,02 |
| Azelnidipine | 1,24 | 1,08 | 0,87 |
| Ambroxol HCl | 1,08 | 1,08 | 1,00 |
| Clorsulon | 1,40 | 1,22 | 0,88 |
| Ibrutinib (PCI-32765) | 1,18 | 1,10 | 0,93 |
| TH-302 | 1,11 | 0,97 | 0,87 |
| Galeterone | 1,13 | 1,14 | 1,01 |
| Prucalopride | 1,22 | 1,13 | 0,93 |
| Paroxetine HCl | 0,94 | 1,01 | 1,08 |
| Cabazitaxel | 0,26 | 0,29 | 1,10 |
| Bepotastine Besilate | 1,11 | 1,04 | 0,94 |
| Acemetacin | 1,23 | 1,45 | 1,18 |
| Alverine Citrate | 1,14 | 1,10 | 0,96 |
| Naloxone HCl | 1,22 | 1,15 | 0,94 |
| Arecoline | 1,14 | 1,28 | 1,12 |
| Resminostat | 0,20 | 0,17 | 0,88 |
| Canagliflozin | 1,28 | 1,15 | 0,90 |
| Dabrafenib (GSK2118436) | 0,73 | 0,84 | 1,15 |
| Cobicistat (GS-9350) | 1,20 | 1,06 | 0,88 |
| Zaltoprofen | 1,06 | 1,11 | 1,04 |
| Bufexamac | 1,12 | 1,11 | 0,99 |
| Fosaprepitant dimeglumine salt | 1,01 | 1,13 | 1,12 |
| Tioxolone | 1,33 | 1,36 | 1,02 |
| Azilsartan Medoxomil | 1,24 | 1,27 | 1,03 |
| Chlorhexidine HCl | 0,52 | 0,21 | 0,40 |
| Noradrenaline bitartrate monohydrate | 1,16 | 1,18 | 1,02 |
| Nilvadipine | 1,30 | 1,24 | 0,96 |
| Dinaciclib (SCH727965) | 0,18 | 0,29 | 1,60 |
| BYL719 | 0,88 | 1,01 | 1,14 |
| S-Ruxolitinib (INCB018424) | 1,16 | 1,14 | 0,98 |
| Pazopanib | 1,22 | 1,28 | 1,05 |
| Lamotrigine | 1,19 | 1,20 | 1,01 |
| Rofecoxib | 1,14 | 1,36 | 1,20 |
| Piracetam | 1,23 | 1,28 | 1,04 |
| Hyoscyamine | 1,17 | 0,94 | 0,80 |
| Homatropine Bromide | 1,16 | 0,98 | 0,84 |
| Ulipristal | 1,27 | 1,07 | 0,84 |
| Oxybutynin chloride | 1,23 | 1,24 | 1,01 |
| Methylthiouracil | 1,28 | 1,04 | 0,81 |
| Butenafine HCl | 1,12 | 1,15 | 1,03 |
| Anagrelide HCl | 1,11 | 1,08 | 0,97 |
| Amitriptyline HCl | 1,23 | 1,08 | 0,88 |
| Triflusal | 1,20 | 0,92 | 0,77 |
| Lithocholic acid | 1,35 | 1,06 | 0,78 |
| Caspofungin Acetate | 1,29 | 1,25 | 0,97 |
| Allylthiourea | 0,93 | 0,99 | 1,07 |
| Hydroxyzine 2HCl | 0,84 | 0,87 | 1,03 |
| Indacaterol Maleate | 1,16 | 1,14 | 0,98 |
| Ornidazole | 1,29 | 1,21 | 0,94 |
| Methenamine | 1,19 | 1,34 | 1,13 |
| Mepivacaine HCl | 1,21 | 0,99 | 0,82 |
| Antipyrine | 1,23 | 1,18 | 0,96 |
| Adrenalone HCl | 0,98 | 0,99 | 1,02 |
| Trifluoperazine 2HCl | 1,18 | 0,85 | 0,72 |
| Ethambutol HCl | 1,05 | 1,04 | 0,99 |
| Dexmedetomidine | 1,23 | 1,08 | 0,88 |
| Avanafil | 0,88 | 0,76 | 0,87 |
| Aclidinium Bromide | 0,94 | 0,87 | 0,92 |
| Moguisteine | 1,26 | 0,97 | 0,77 |
| Dexamethasone Acetate | 1,24 | 1,16 | 0,93 |
| Milnacipran HCl | 1,10 | 0,90 | 0,82 |
| Ethynodiol diacetate | 1,14 | 0,95 | 0,84 |
| Atomoxetine HCl | 1,14 | 0,99 | 0,87 |
| Azatadine dimaleate | 1,05 | 0,98 | 0,93 |
| Meptazinol HCl | 1,10 | 0,85 | 0,77 |
| Doxycycline HCl | 0,80 | 0,76 | 0,95 |
| Foscarnet Sodium | 1,27 | 1,17 | 0,92 |
| Sodium Picosulfate | 0,90 | 1,05 | 1,17 |
| Diphemanil Methylsulfate | 0,96 | 1,07 | 1,11 |
| Nadifloxacin | 1,18 | 1,05 | 0,89 |
| Trimethoprim | 1,11 | 1,24 | 1,12 |
| Darifenacin HBr | 1,03 | 0,94 | 0,91 |
| Sertaconazole nitrate | 1,21 | 0,92 | 0,76 |
| Betahistine 2HCl | 1,20 | 1,00 | 0,84 |
| (+,-)-Octopamine HCl | 1,03 | 1,07 | 1,03 |
| Fexofenadine HCl | 1,03 | 1,00 | 0,98 |
| Pentamidine | 0,93 | 0,84 | 0,90 |
| Tazobactam | 1,26 | 1,07 | 0,85 |
| Tolcapone | 0,83 | 0,83 | 0,99 |
| Vitamin D2 | 0,94 | 0,90 | 0,96 |
| Pidotimod | 1,23 | 1,12 | 0,91 |
| Biotin (Vitamin B7) | 1,16 | 1,15 | 0,98 |
| Entacapone | 1,12 | 1,08 | 0,97 |
| Tylosin tartrate | 1,17 | 1,08 | 0,92 |
| Brinzolamide | 1,01 | 1,15 | 1,14 |
| Ropinirole HCl | 0,90 | 1,11 | 1,23 |
| Moclobemide (Ro 111163) | 0,90 | 0,95 | 1,06 |
| Mirabegron | 0,98 | 1,03 | 1,05 |
| Beclomethasone dipropionate | 1,30 | 1,27 | 0,98 |
| Probenecid | 1,05 | 1,07 | 1,02 |
| Doxapram HCl | 1,17 | 1,06 | 0,90 |
| Pyridoxine HCl | 1,34 | 1,22 | 0,90 |
| Sulfamerazine | 1,22 | 1,21 | 0,99 |
| Estradiol valerate | 1,19 | 1,13 | 0,95 |
| Benztropine mesylate | 1,12 | 1,04 | 0,93 |
| Carbenicillin disodium | 1,14 | 1,02 | 0,90 |
| Azlocillin sodium salt | 1,12 | 1,05 | 0,94 |
| Pergolide mesylate | 1,10 | 0,91 | 0,83 |
| Acebutolol HCl | 1,15 | 1,00 | 0,86 |
| Atovaquone | 1,37 | 1,28 | 0,93 |
| Procaine HCl | 0,92 | 0,96 | 1,05 |
| Dibucaine HCl | 0,96 | 1,04 | 1,08 |
| Vitamin C | 1,29 | 1,17 | 0,90 |
| Sulfamethazine | 1,22 | 1,19 | 0,98 |
| Articaine HCl | 1,19 | 1,19 | 1,00 |
| Altrenogest | 1,19 | 1,11 | 0,93 |
| Eletriptan HBr | 1,15 | 1,04 | 0,91 |
| Azacyclonol | 1,06 | 1,01 | 0,95 |
| Cabozantinib malate (XL184) | 1,07 | 0,88 | 0,82 |
| Ampiroxicam | 0,97 | 1,02 | 1,06 |
| Etravirine (TMC125) | 1,32 | 1,29 | 0,98 |
| Homatropine Methylbromide | 1,19 | 1,16 | 0,98 |
| Methazolamide | 1,40 | 1,14 | 0,81 |
| Sulfathiazole | 1,17 | 1,19 | 1,01 |
| Sodium salicylate | 1,36 | 1,37 | 1,00 |
| Gliquidone | 1,18 | 1,19 | 1,01 |
| Ampicillin sodium | 1,25 | 1,28 | 1,03 |
| Flumequine | 1,17 | 1,21 | 1,04 |
| Reboxetine mesylate | 1,30 | 1,22 | 0,94 |
| Sitagliptin phosphate monohydrate | 1,32 | 1,15 | 0,88 |
| Desloratadine | 1,30 | 1,18 | 0,91 |
| Norethindrone | 1,32 | 1,07 | 0,81 |
| Dinitolmide | 1,28 | 1,25 | 0,97 |
| Chlorzoxazone | 1,33 | 1,21 | 0,91 |
| Carbimazole | 1,11 | 1,04 | 0,94 |
| Ropivacaine HCl | 1,18 | 1,09 | 0,92 |
| Tinidazole | 1,13 | 1,15 | 1,02 |
| Mequinol | 1,19 | 1,33 | 1,12 |
| Loxapine Succinate | 1,15 | 1,10 | 0,95 |
| Halcinonide | 1,34 | 1,21 | 0,90 |
| Estradiol Benzoate | 1,27 | 1,23 | 0,97 |
| Timolol Maleate | 1,22 | 1,27 | 1,04 |
| Olsalazine Sodium | 1,30 | 1,12 | 0,86 |
| Clopidol | 1,24 | 1,08 | 0,87 |
| Chlortetracycline HCl | 1,45 | 1,21 | 0,84 |
| Valdecoxib | 1,05 | 1,01 | 0,96 |
| Sodium Nitroprusside | 1,18 | 0,93 | 0,79 |
| Guanidine HCl | 1,10 | 1,08 | 0,98 |
| Mefenamic Acid | 1,21 | 1,14 | 0,95 |
| Flumethasone | 1,11 | 1,11 | 0,99 |
| Dexlansoprazole | 1,08 | 1,21 | 1,11 |
| Dicloxacillin Sodium | 1,26 | 1,23 | 0,98 |
| Tolazoline HCl | 1,21 | 1,10 | 0,90 |
| Nafcillin Sodium | 1,06 | 0,92 | 0,87 |
| Bacitracin | 1,22 | 1,01 | 0,83 |
| Bezafibrate | 1,19 | 1,07 | 0,90 |
| Valganciclovir HCl | 1,01 | 0,94 | 0,93 |
| Erythromycin Ethylsuccinate | 0,98 | 0,98 | 0,99 |
| Griseofulvin | 0,98 | 0,96 | 0,98 |
| Ticagrelor | 0,99 | 1,02 | 1,03 |
| Halobetasol Propionate | 1,04 | 1,06 | 1,01 |
| Esmolol HCl | 1,13 | 1,06 | 0,94 |
| Desvenlafaxine Succinate | 1,12 | 1,10 | 0,98 |
| Sodium Phenylbutyrate | 1,13 | 1,14 | 1,01 |
| Tetrahydrozoline HCl | 1,06 | 0,99 | 0,93 |
| Azithromycin Dihydrate | 0,98 | 1,14 | 1,16 |
| Penicillin G Sodium | 1,08 | 1,10 | 1,02 |
| Nabumetone | 0,88 | 1,05 | 1,19 |
| Levobupivacaine HCl | 1,07 | 1,05 | 0,98 |
| Decamethonium Bromide | 1,08 | 1,00 | 0,93 |
| Triamterene | 0,72 | 1,01 | 1,41 |
| Fenspiride HCl | 1,05 | 0,98 | 0,94 |
| Voglibose | 1,02 | 1,23 | 1,21 |
| Desvenlafaxine | 1,11 | 1,06 | 0,96 |
| Troxipide | 1,05 | 1,12 | 1,06 |
| Toltrazuril | 1,08 | 0,97 | 0,90 |
| Ampicillin Trihydrate | 1,21 | 1,10 | 0,91 |
| Benzoic Acid | 1,16 | 1,04 | 0,90 |
| Sertraline HCl | 1,11 | 0,96 | 0,86 |
| Ronidazole | 0,98 | 1,10 | 1,13 |
| Sodium 4-Aminosalicylate | 1,19 | 0,98 | 0,82 |
| Sulfacetamide Sodium | 1,06 | 1,11 | 1,05 |
| Pramoxine HCl | 1,00 | 0,87 | 0,87 |
| Eprosartan Mesylate | 1,09 | 1,16 | 1,07 |
| Triclabendazole | 1,13 | 1,06 | 0,94 |
| Clorprenaline HCl | 1,14 | 1,14 | 1,00 |
| Pheniramine Maleate | 1,23 | 1,05 | 0,85 |
| Amfenac Sodium Monohydrate | 1,13 | 0,96 | 0,84 |
| Benzethonium Chloride | 0,64 | 0,40 | 0,63 |
| Spironolactone | 1,06 | 1,06 | 1,00 |
| Vitamin D3 | 1,02 | 1,04 | 1,02 |
| Sodium Nitrite | 0,93 | 1,10 | 1,19 |
| Spiramycin | 1,07 | 1,09 | 1,01 |
| Difluprednate | 1,15 | 0,97 | 0,85 |
| Closantel Sodium | 1,00 | 1,11 | 1,11 |
| Histamine 2HCl | 0,99 | 1,13 | 1,15 |
| Carprofen | 1,23 | 1,04 | 0,84 |
| Estradiol Cypionate | 0,93 | 1,20 | 1,29 |
| Penfluridol | 1,57 | 1,04 | 0,67 |
| Doxofylline | 1,20 | 1,10 | 0,92 |
| Retapamulin | 1,21 | 1,45 | 1,19 |
| Escitalopram Oxalate | 0,97 | 1,15 | 1,19 |
| Zinc Pyrithione | 0,01 | 0,02 | 1,84 |
| Lomerizine HCl | 0,91 | 1,15 | 1,26 |
| Droperidol | 1,11 | 1,09 | 0,97 |
| Closantel | 1,16 | 1,18 | 1,02 |
| Sulconazole Nitrate | 1,08 | 1,07 | 0,99 |
| Dropropizine | 1,20 | 1,21 | 1,00 |
| Bisacodyl | 1,17 | 1,21 | 1,04 |
| Ethamsylate | 1,30 | 1,30 | 1,00 |
| Benzydamine HCl | 1,26 | 1,23 | 0,98 |
| Methyclothiazide | 1,15 | 1,22 | 1,06 |
| Guanabenz Acetate | 1,27 | 1,26 | 0,99 |
| Propranolol HCl | 1,31 | 1,23 | 0,94 |
| Levobetaxolol HCl | 1,14 | 1,34 | 1,17 |
| Dydrogesterone | 1,25 | 1,14 | 0,91 |
| Clofazimine | 1,31 | 1,20 | 0,92 |
| Tilmicosin | 1,17 | 1,16 | 0,99 |
| Cyclizine 2HCl | 1,22 | 1,19 | 0,98 |
| Chlorpropamide | 1,08 | 1,07 | 0,99 |
| Cyclobenzaprine HCl | 1,22 | 1,11 | 0,91 |
| Diphenidol HCl | 1,28 | 1,45 | 1,14 |
| Uracil | 1,09 | 0,90 | 0,82 |
| Cyclandelate | 1,07 | 0,99 | 0,93 |
| Antazoline HCl | 0,98 | 0,94 | 0,96 |
| Chlorocresol | 1,15 | 0,95 | 0,83 |
| Bosentan | 1,21 | 0,84 | 0,69 |
| Oxaprozin | 1,13 | 0,93 | 0,83 |
| Prucalopride Succinat | 1,15 | 0,96 | 0,84 |
| Diacerein | 1,22 | 1,12 | 0,92 |
| Cyromazine | 1,03 | 0,95 | 0,92 |
| Ospemifene | 1,00 | 0,85 | 0,85 |
| Promethazine HCl | 1,10 | 0,87 | 0,78 |
| Climbazole | 0,96 | 0,83 | 0,87 |
| Cinchophen | 0,95 | 0,97 | 1,02 |
| Tolperisone HCl | 1,14 | 0,82 | 0,72 |
| Benzocaine | 0,99 | 0,83 | 0,84 |
| Benzbromarone | 1,10 | 0,85 | 0,77 |
| Zoxazolamine | 1,11 | 0,80 | 0,72 |
| Bromfenac Sodium | 0,94 | 0,84 | 0,89 |
| Flufenamic acid | 1,13 | 0,86 | 0,76 |
| Teriflunomide | 0,85 | 0,91 | 1,06 |
| Anidulafungin (LY303366) | 0,99 | 1,05 | 1,07 |
| Procainamide HCl | 1,14 | 1,21 | 1,06 |
| Mezlocillin Sodium | 0,91 | 1,12 | 1,23 |
| Betamipron | 1,13 | 0,94 | 0,83 |
| Florfenicol | 1,41 | 1,20 | 0,85 |
| Montelukast Sodium | 1,06 | 0,97 | 0,91 |
| Piperacillin Sodium | 1,10 | 0,89 | 0,81 |
| Phenazopyridine HCl | 1,10 | 0,94 | 0,86 |
| Sulfamethoxypyridazine | 0,90 | 0,75 | 0,83 |
| Vinorelbine Tartrate | 0,11 | 0,15 | 1,31 |
| Coumarin | 1,06 | 1,13 | 1,07 |
| Micafungin Sodium | 1,07 | 1,01 | 0,94 |
| Meclofenamate Sodium | 1,11 | 1,08 | 0,97 |
| Nicardipine HCl | 1,01 | 1,00 | 0,98 |
| Chlorquinaldol | 0,72 | 0,76 | 1,06 |
| Furaltadone HCl | 1,09 | 1,03 | 0,95 |
| Dirithromycin | 0,97 | 1,02 | 1,05 |
| Mevastatin | 0,53 | 0,68 | 1,29 |
| Doxylamine Succinate | 0,97 | 0,99 | 1,02 |
| Epinastine HCl | 1,14 | 0,94 | 0,83 |
| Oxiracetam | 1,12 | 1,02 | 0,90 |
| Choline Chloride | 1,02 | 1,07 | 1,04 |
| Chloroambucil | 0,97 | 1,14 | 1,18 |
| Salmeterol Xinafoate | 0,99 | 1,13 | 1,14 |
| Nifuroxazide | 0,58 | 0,60 | 1,02 |
| Broxyquinoline | 0,64 | 0,67 | 1,04 |
| Isosorbide | 0,78 | 0,92 | 1,18 |
| Valnemulin HCl | 1,12 | 1,15 | 1,03 |
| Mexiletine HCl | 0,82 | 1,01 | 1,24 |
| Cetrimonium Bromide (CTAB) | 0,08 | 0,05 | 0,61 |
| Buspirone HCl | 1,02 | 0,95 | 0,93 |
| Rotigotine | 0,98 | 1,00 | 1,02 |
| Cetylpyridinium Chloride | 0,07 | 0,05 | 0,64 |
| MetoclopraMide HCl | 1,12 | 1,03 | 0,92 |
| Mupirocin | 1,19 | 1,21 | 1,01 |
| Penciclovir | 1,10 | 0,97 | 0,87 |
| Ethacridine lactate monohydrate | 0,40 | 0,47 | 1,16 |
| Cysteamine HCl | 1,17 | 0,98 | 0,84 |
| Liothyronine Sodium | 1,16 | 0,89 | 0,76 |
| Fidaxomicin | 0,84 | 0,56 | 0,67 |
| Deoxycorticosterone acetate | 1,00 | 0,95 | 0,94 |
| Luliconazole | 1,08 | 0,95 | 0,88 |
| Carteolol HCl | 1,05 | 0,97 | 0,92 |
| Sulfaguanidine | 0,98 | 0,99 | 1,01 |
| Digoxin | 0,04 | 0,09 | 2,26 |
| (R)-(+)-Atenolol | 0,88 | 1,01 | 1,15 |
| Tiratricol | 0,83 | 1,05 | 1,26 |
| Bemegride | 1,11 | 1,02 | 0,92 |
| Clofibric Acid | 1,06 | 0,97 | 0,91 |
| Amoxapine | 1,20 | 0,85 | 0,71 |
| Fluorometholone Acetate | 1,26 | 0,89 | 0,71 |
| Serotonin HCl | 1,27 | 0,95 | 0,75 |
| Tamibarotene | 1,18 | 1,12 | 0,95 |
| Demeclocycline HCl | 1,31 | 1,21 | 0,92 |
| Trometamol | 1,22 | 1,28 | 1,05 |
| Labetalol HCl | 1,28 | 1,33 | 1,04 |
| Anisotropine Methylbromide | 1,13 | 1,17 | 1,03 |
| Domiphen Bromide | 0,41 | 0,25 | 0,61 |
| Aminothiazole | 1,24 | 1,21 | 0,98 |
| Chromocarb | 1,17 | 1,25 | 1,07 |
| Azaperone | 1,15 | 1,12 | 0,97 |
| Oxybuprocaine HCl | 1,09 | 1,04 | 0,95 |
| Tranylcypromine (2-PCPA) HCl | 1,28 | 1,15 | 0,90 |
| Ebastine | 1,30 | 0,96 | 0,74 |
| Nelfinavir Mesylate | 1,44 | 1,08 | 0,75 |
| Benzthiazide | 1,19 | 1,07 | 0,90 |
| Salicylic acid | 1,12 | 1,18 | 1,05 |
| Bithionol | 1,43 | 1,09 | 0,76 |
| Metaproterenol Sulfate | 1,23 | 1,14 | 0,93 |
| Procyclidine HCl | 1,11 | 1,07 | 0,96 |
| Noscapine HCl | 1,18 | 1,08 | 0,91 |
| Imipramine HCl | 1,25 | 1,21 | 0,97 |
| Diperodon HCl | 1,23 | 1,10 | 0,89 |
| Mefloquine HCl | 1,34 | 0,97 | 0,72 |
| (+)-Camphor | 1,17 | 1,21 | 1,04 |
| Fenofibric acid | 1,26 | 1,14 | 0,91 |
| Carbadox | 1,18 | 1,22 | 1,03 |
| Triclosan | 1,07 | 1,02 | 0,95 |
| Bronopol | 0,85 | 0,67 | 0,79 |
| Metaraminol Bitartrate | 1,04 | 1,13 | 1,08 |
| Ractopamine HCl | 1,12 | 1,26 | 1,12 |
| Phthalylsulfacetamide | 1,11 | 1,19 | 1,08 |
| Proadifen HCl | 1,09 | 1,13 | 1,04 |
| Isoxicam | 1,11 | 1,07 | 0,96 |
| Eltrombopag | 1,10 | 1,11 | 1,01 |
| Cefotaxime sodium | 1,02 | 1,13 | 1,11 |
| Furazolidone | 0,96 | 0,96 | 1,00 |
| Diphenylpyraline HCl | 1,11 | 1,23 | 1,11 |
| Trihexyphenidyl hydrochloride | 1,14 | 1,02 | 0,89 |
| Bucetin | 1,03 | 0,91 | 0,88 |
| Meticrane | 1,08 | 1,16 | 1,08 |
| Terfenadine | 1,01 | 1,09 | 1,08 |
| Carbenoxolone Sodium | 1,02 | 0,93 | 0,91 |
| Pyrilamine Maleate | 1,09 | 1,07 | 0,98 |
| Nifenazone | 1,07 | 0,91 | 0,85 |
| 6-Mercaptopurine (6-MP) Monohydrate | 0,55 | 0,32 | 0,58 |
| Chloroxylenol | 1,04 | 1,07 | 1,03 |
| Idramantone | 1,15 | 0,94 | 0,81 |
| Disopyramide Phosphate | 1,21 | 1,22 | 1,01 |
| Trimetazidine dihydrochloride | 1,01 | 0,87 | 0,85 |
| Carsalam | 1,01 | 0,84 | 0,83 |
| Moxalactam Disodium | 1,11 | 1,25 | 1,12 |
| Tolazamide | 1,02 | 1,04 | 1,02 |
| Nicotine Ditartrate | 1,07 | 1,15 | 1,07 |
| Difloxacin HCl | 1,08 | 1,10 | 1,02 |
| Oxeladin Citrate | 1,07 | 1,00 | 0,93 |
| Vinblastine sulfate | 0,13 | 0,20 | 1,52 |
| Citric acid trilithium salt tetrahydrate | 0,98 | 0,97 | 0,99 |
| i-Inositol | 0,99 | 0,91 | 0,92 |
| Ethoxzolamide | 1,52 | 1,37 | 0,90 |
| Urethane | 1,07 | 0,91 | 0,84 |
| Carzenide | 1,14 | 0,97 | 0,85 |
| Nalmefene HCl | 1,14 | 1,20 | 1,06 |
| Tacrine HCl | 1,05 | 1,11 | 1,06 |
| Dicyclomine HCl | 1,01 | 1,19 | 1,18 |
| Fosfomycin Tromethamine | 1,04 | 1,07 | 1,02 |
| Pasiniazid | 1,07 | 0,98 | 0,92 |
| Acetazolamide | 1,00 | 0,92 | 0,92 |
| DEET | 1,05 | 1,08 | 1,03 |
| Iopamidol | 1,05 | 0,97 | 0,93 |
| Isoetharine Mesylate | 1,23 | 1,24 | 1,01 |
| Xylitol | 1,02 | 0,94 | 0,93 |
| Citiolone | 1,14 | 0,85 | 0,74 |
| Nialamide | 1,13 | 1,18 | 1,04 |
| Pimozide | 1,14 | 1,14 | 1,00 |
| Thioridazine HCl | 1,05 | 1,11 | 1,05 |
| Bephenium Hydroxynaphthoate | 1,06 | 1,17 | 1,10 |
| Procodazole | 1,05 | 1,02 | 0,97 |
| 17-Hydroxyprogesterone | 0,97 | 1,00 | 1,03 |
| Dehydroacetic acid | 1,15 | 1,17 | 1,02 |
| Methylene Blue | 0,17 | 0,33 | 1,95 |
| Mepiroxol | 1,20 | 1,27 | 1,06 |
| Aminoguanidine (hydrochloride) | 1,02 | 1,00 | 0,98 |
| Cloxiquine | 0,93 | 0,78 | 0,84 |
| Pentoxifylline | 1,09 | 1,15 | 1,05 |
| Carbachol | 1,23 | 1,16 | 0,94 |
| Mepenzolate Bromide | 1,18 | 1,22 | 1,04 |
| Brucine | 1,07 | 1,24 | 1,16 |
| Sodium 4-aminohippurate Hydrate | 0,98 | 1,14 | 1,16 |
| Aceglutamide | 1,10 | 1,06 | 0,96 |
| Ethylparaben | 1,01 | 1,05 | 1,04 |
| Nitrofurantoin | 1,02 | 1,09 | 1,07 |
| Mesoridazine Besylate | 1,40 | 1,26 | 0,90 |
| Azelaic acid | 1,07 | 1,14 | 1,07 |
| Danthron | 1,08 | 1,02 | 0,94 |
| Piromidic Acid | 1,26 | 1,32 | 1,05 |
| Glafenine HCl | 1,23 | 1,27 | 1,03 |
| Aceclidine HCl | 1,23 | 1,21 | 0,98 |
| Clofoctol | 1,22 | 1,29 | 1,05 |
| Trimipramine Maleate | 1,22 | 1,17 | 0,96 |
| Acetylleucine | 1,10 | 1,12 | 1,01 |
| Fenbufen | 1,12 | 1,08 | 0,96 |
| Pantoprazole sodium | 1,10 | 1,08 | 0,99 |
| Dehydrocholic acid | 1,22 | 1,24 | 1,02 |
| Nandrolone | 1,21 | 1,07 | 0,89 |
| Lidocaine hydrochloride | 1,09 | 1,12 | 1,02 |
| Sulfabenzamide | 1,26 | 1,07 | 0,85 |
| Succinylsulfathiazole | 1,14 | 1,09 | 0,95 |
| Lercanidipine (hydrochloride) | 1,01 | 1,10 | 1,09 |
| 2,2′-Dihydroxy-4-methoxybenzophenone | 1,09 | 1,08 | 0,99 |
| Itopride hydrochloride | 1,00 | 1,02 | 1,03 |
| Prochlorperazine dimaleate salt | 1,26 | 1,10 | 0,87 |
| Brexpiprazole | 1,16 | 1,00 | 0,86 |
| Atipamezole hydrochloride | 1,20 | 1,02 | 0,85 |
| Diethylcarbamazine (citrate) | 1,25 | 1,23 | 0,99 |
| Glycopyrrolate | 1,05 | 1,04 | 0,99 |
| Procaine | 1,10 | 0,97 | 0,88 |
| Terpin (hydrate) | 1,16 | 1,09 | 0,94 |
| Docusate Sodium | 1,19 | 1,09 | 0,92 |
| Benzyl benzoate | 1,08 | 1,03 | 0,96 |
| Diflunisal | 1,00 | 0,92 | 0,91 |
| Cefuroxime sodium | 1,00 | 0,96 | 0,96 |
| Hexachlorophene | 1,15 | 0,92 | 0,80 |
| Lesinurad | 1,08 | 0,91 | 0,85 |
| Atipamezole | 1,00 | 0,99 | 0,99 |
| Diiodohydroxyquinoline | 1,25 | 1,26 | 1,01 |
| Tiagabine hydrochloride | 1,05 | 0,80 | 0,76 |
| Benzocaine hydrochloride | 1,00 | 0,96 | 0,96 |
| Tyloxapol | 1,15 | 0,96 | 0,83 |
| Amodiaquin (dihydrochloride dihydrate) | 0,95 | 0,83 | 0,87 |
| Benzyl alcohol | 0,99 | 0,84 | 0,85 |
| Mebendazole | 0,11 | 0,12 | 1,16 |
| 5,5-Dimethyloxazolidine-2,4-dione | 1,04 | 0,86 | 0,83 |
| Isosorbide Mononitrate | 1,04 | 0,91 | 0,87 |
| Tedizolid Phosphate | 1,44 | 0,99 | 0,69 |
| Etoricoxib | 1,01 | 0,90 | 0,90 |
| DL-Panthenol | 1,23 | 1,23 | 1,01 |
| Atazanavir | 0,89 | 0,79 | 0,89 |
| Etonogestrel | 0,99 | 1,00 | 1,01 |
| Resorcinol | 1,04 | 1,07 | 1,03 |
| Nitroxoline | 0,12 | 0,15 | 1,22 |
| 5-Chloro-8-hydroxy-7-iodoquinoline | 1,04 | 1,04 | 1,01 |
| Dapson | 0,96 | 0,82 | 0,85 |
| Alcaftadine | 0,90 | 0,95 | 1,05 |
| Sodium sulfadiazine | 0,99 | 0,95 | 0,96 |
| Zolpidem | 1,03 | 0,84 | 0,81 |
| Sulisobenzone | 0,94 | 0,84 | 0,90 |
| Fluphenazine (dihydrochloride) | 1,37 | 1,24 | 0,90 |
| Fusidate Sodium | 0,85 | 0,94 | 1,10 |
| Hydroxyprogesterone caproate | 1,08 | 1,16 | 1,07 |
| Hydroquinone | 1,31 | 1,36 | 1,05 |
| Chlormadinone acetate | 1,10 | 1,09 | 1,00 |
| Acetohydroxamic acid | 1,12 | 1,14 | 1,01 |
| Modafinil | 1,01 | 0,95 | 0,94 |
| Ethosuximide | 1,07 | 0,91 | 0,85 |
| Cyproheptadine hydrochloride | 1,11 | 0,99 | 0,89 |
| Armodafinil | 1,01 | 0,98 | 0,97 |
| Sulpiride | 1,02 | 0,88 | 0,86 |
| Halothane | 1,26 | 1,30 | 1,03 |
| Molsidomine | 0,99 | 1,00 | 1,01 |
| Tiagabine | 0,88 | 1,04 | 1,19 |
| Triacetin | 1,20 | 1,24 | 1,03 |
| Cephalothin | 1,13 | 1,12 | 0,98 |
| Gallic acid | 0,90 | 1,12 | 1,24 |
| Nandrolone decanoate | 1,02 | 1,08 | 1,06 |
| (+/-)-Sulfinpyrazone | 1,00 | 1,01 | 1,00 |
| Teneligliptin hydrobromide | 1,08 | 0,86 | 0,80 |
| Ciclesonide | 1,04 | 0,98 | 0,94 |
| Parecoxib | 0,98 | 1,00 | 1,02 |
| Hexylresorcinol | 1,24 | 1,46 | 1,18 |
| Rebeprazole sodium | 1,11 | 1,04 | 0,93 |
| Gluconolactone | 0,95 | 1,14 | 1,20 |
| Butamben | 1,14 | 1,19 | 1,04 |
| Cefazolin Sodium | 1,07 | 1,18 | 1,10 |
| Levofloxacin hydrate | 0,98 | 1,23 | 1,25 |
| Dextromethorphan (hydrobromide hydrate) | 1,05 | 1,05 | 1,00 |
| Chlorotrianisene | 1,00 | 1,15 | 1,15 |
| Prasugrel Hydrochloride | 1,38 | 1,44 | 1,04 |
| Cefmenoxime hydrochloride | 1,18 | 1,09 | 0,92 |
| Eslicarbazepine Acetate | 1,03 | 1,02 | 0,99 |
| Piperazine | 1,32 | 1,24 | 0,94 |
| Sivelestat sodium | 0,81 | 0,80 | 0,98 |
| Fluoxymesterone | 1,04 | 1,23 | 1,18 |
| Butylparaben | 1,21 | 1,18 | 0,98 |
| Cefixime | 1,31 | 1,23 | 0,94 |
| Folic acid | 1,18 | 1,17 | 0,99 |
| Fenoldopam (mesylate) | 1,20 | 1,23 | 1,03 |
| Diazoxide | 1,09 | 1,19 | 1,10 |
| Desogestrel | 1,29 | 1,14 | 0,89 |
| Dantrolene sodium | 1,14 | 1,15 | 1,01 |
| Hydroquinidine | 1,28 | 1,18 | 0,92 |
| Povidone iodine | 1,28 | 1,21 | 0,94 |
| Pexmetinib (ARRY-614) | 0,96 | 0,82 | 0,85 |
| Epacadostat (INCB024360) | 1,23 | 1,12 | 0,91 |
| Perampanel | 1,18 | 1,15 | 0,98 |
| Tofacitinib (CP-690550) Citrate | 1,19 | 1,18 | 0,99 |
| Tepotinib (EMD 1214063) | 1,09 | 0,98 | 0,90 |
| Motolimod (VTX-2337) | 0,96 | 0,77 | 0,80 |
| Edoxaban | 1,13 | 1,14 | 1,01 |
| Lomitapide Mesylate | 0,83 | 0,53 | 0,63 |
| Salirasib | 1,25 | 1,10 | 0,88 |
| Terazosin | 1,05 | 1,04 | 1,00 |
| Afatinib (BIBW2992) Dimaleate | 0,32 | 0,25 | 0,78 |
| Ozanimod (RPC1063) | 0,97 | 0,90 | 0,93 |
| Deoxycholic acid | 1,01 | 0,96 | 0,95 |
| Fingolimod (FTY720) HCl | 0,94 | 0,67 | 0,71 |
| Cilengitide | 0,83 | 0,81 | 0,98 |
| BAF312 (Siponimod) | 1,09 | 0,93 | 0,86 |
| CO-1686 (AVL-301) | 1,09 | 1,02 | 0,93 |
| LEE011 | 0,95 | 0,79 | 0,84 |
| Cerdulatinib (PRT062070, PRT2070) | 1,16 | 0,94 | 0,81 |
| Dovitinib (TKI258) Lactate | 0,46 | 0,56 | 1,20 |
| Protirelin | 0,96 | 1,00 | 1,03 |
| Pexidartinib (PLX3397) | 0,94 | 0,95 | 1,01 |
| Napabucasin | 0,00 | 0,01 | 3,56 |
| Escin | 1,02 | 1,04 | 1,01 |
| Tacrolimus (FK506) | 1,05 | 0,96 | 0,91 |
| LDK378 | 0,90 | 0,97 | 1,08 |
| Idasanutlin (RG-7388) | 1,14 | 1,00 | 0,88 |
| AZD9291 | 0,79 | 0,82 | 1,04 |
| Uprosertib (GSK2141795) | 0,64 | 0,65 | 1,03 |
| Lomitapide | 0,80 | 0,60 | 0,75 |
| Elacridar (GF120918) | 1,00 | 1,01 | 1,00 |
| Loxoprofen | 0,96 | 0,89 | 0,93 |
| CB1954 | 0,92 | 0,99 | 1,08 |
| Ripasudil (K-115) | 1,21 | 1,28 | 1,06 |
| Oxybenzone | 0,97 | 0,99 | 1,02 |
| Pimecrolimus | 1,06 | 1,06 | 1,00 |
| EPZ-6438 | 1,06 | 0,97 | 0,92 |
| Losmapimod (GW856553X) | 0,83 | 0,91 | 1,09 |
| Rilpivirine | 0,98 | 0,95 | 0,97 |
| Trelagliptin | 0,92 | 1,01 | 1,10 |
| Pilaralisib (XL147) | 0,88 | 0,93 | 1,05 |
| Emricasan | 1,02 | 0,98 | 0,96 |
| Sildenafil Mesylate | 0,96 | 0,96 | 1,00 |
| Eliglustat Tartrate | 0,99 | 1,13 | 1,15 |
| Entrectinib (RXDX-101) | 0,78 | 0,69 | 0,88 |
| Guanfacine Hydrochloride | 0,98 | 0,92 | 0,93 |
| MEK162 (ARRY-162, ARRY-438162) | 0,96 | 0,88 | 0,92 |
| Batimastat (BB-94) | 0,84 | 0,81 | 0,97 |
| Alvelestat (AZD9668) | 0,84 | 0,90 | 1,08 |
| Tasisulam | 0,78 | 0,72 | 0,92 |
| Afuresertib (GSK2110183) | 0,74 | 0,79 | 1,07 |
| Voxtalisib (XL765, SAR245409) | 0,59 | 0,48 | 0,82 |
| Sunitinib | 0,68 | 0,62 | 0,91 |
| Efavirenz | 0,90 | 1,01 | 1,12 |
| Ulixertinib (BVD-523, VRT752271) | 1,02 | 0,74 | 0,72 |
| Rocilinostat (ACY-1215) | 1,11 | 1,08 | 0,98 |
| D panthenol | 1,03 | 0,95 | 0,92 |
| Birinapant | 0,59 | 0,25 | 0,42 |
| Marimastat(BB-2516) | 0,88 | 0,74 | 0,84 |
| KPT-330 | 0,09 | 0,08 | 0,83 |
| Poziotinib (HM781-36B) | 0,86 | 0,79 | 0,92 |
| GS-9973 | 1,05 | 0,88 | 0,84 |
| Defactinib (VS-6063, PF-04554878) | 0,93 | 0,89 | 0,95 |
| Dasatinib Monohydrate | 0,44 | 0,36 | 0,83 |
| Vitamin E | 1,06 | 1,14 | 1,08 |
| Tenofovir Alafenamide (GS-7340) | 0,97 | 0,70 | 0,73 |
| Otenabant (CP-945598) HCl | 0,90 | 1,10 | 1,22 |
| ArbinoxaMine Maleate | 1,01 | 1,02 | 1,01 |
| Embelin | 1,06 | 1,19 | 1,12 |
| Ilomastat (GM6001, Galardin) | 1,05 | 0,99 | 0,94 |
| Vidofludimus | 0,85 | 0,75 | 0,88 |
| Aloxistatin | 1,09 | 1,15 | 1,06 |
| Ledipasvir (GS5885) | 0,91 | 0,91 | 1,00 |
| Obeticholic Acid | 1,01 | 1,05 | 1,04 |
| Erlotinib | 0,90 | 0,74 | 0,82 |
| Rivastigmine | 1,07 | 1,10 | 1,02 |
| Oltipraz | 1,18 | 1,09 | 0,93 |
| Empagliflozin (BI 10773) | 1,24 | 1,08 | 0,87 |
| Saxagliptin hydrate | 1,10 | 1,01 | 0,91 |
| IPI-145 (INK1197) | 0,91 | 1,01 | 1,11 |
| LY2835219 | 0,61 | 0,70 | 1,15 |
| Suvorexant (MK-4305) | 1,11 | 1,02 | 0,92 |
| Sorafenib | 0,86 | 0,72 | 0,83 |
| Filgotinib (GLPG0634) | 0,90 | 1,01 | 1,12 |
| LCZ696 | 1,06 | 1,06 | 1,00 |
| Docetaxel Trihydrate | 0,15 | 0,26 | 1,70 |
| Apremilast (CC-10004) | 1,08 | 1,12 | 1,04 |
| Sodium butyrate | 1,10 | 1,20 | 1,10 |
| Salbutamol Sulfate | 0,84 | 1,35 | 1,61 |
| Radotinib | 1,00 | 0,87 | 0,87 |
| Olmutinib (HM61713, BI 1482694) | 0,96 | 0,87 | 0,91 |
| Bivalirudin Trifluoroacetate | 1,03 | 0,75 | 0,73 |
| Nafarelin Acetate | 1,17 | 0,73 | 0,63 |
| Granisetron HCl | 0,93 | 0,69 | 0,74 |
| Cephalexin | 0,97 | 0,76 | 0,79 |
| Tranexamic Acid | 0,99 | 1,05 | 1,06 |
| Cobimetinib (GDC-0973, RG7420) | 1,07 | 0,58 | 0,54 |
| Taurine | 1,04 | 0,90 | 0,86 |
| Tobramycin | 0,98 | 0,87 | 0,89 |
| Riociguat (BAY 63-2521) | 0,96 | 0,76 | 0,79 |
| Eptifibatide Acetate | 1,01 | 0,87 | 0,85 |
| Palbociclib (PD-0332991) HCl | 0,75 | 0,63 | 0,84 |
| Heparin sodium | 0,96 | 0,94 | 0,98 |
| Perindopril Erbumine | 0,99 | 0,91 | 0,92 |
| D-glutamine | 1,04 | 0,97 | 0,93 |
| ABT-199 (GDC-0199) | 0,86 | 0,82 | 0,96 |
| Clindamycin Phosphate | 0,92 | 0,95 | 1,03 |
| Methacycline HCl | 1,06 | 1,15 | 1,08 |
| Molidustat (BAY 85-3934) | 0,99 | 0,81 | 0,82 |
| Lypressin Acetate | 1,08 | 0,78 | 0,73 |
| Pemetrexed | 0,45 | 0,44 | 0,99 |
| Biapenem | 0,96 | 0,76 | 0,79 |
| Palbociclib (PD0332991) Isethionate | 0,65 | 0,42 | 0,65 |
| Disodium Cromoglycate | 0,91 | 0,90 | 0,99 |
| Macitentan | 0,95 | 0,85 | 0,89 |
| Lisinopril | 0,90 | 1,07 | 1,18 |
| Lomefloxacin HCl | 0,84 | 1,00 | 1,19 |
| Mitomycin C | 0,13 | 0,12 | 0,92 |
| Octreotide Acetate | 0,87 | 0,83 | 0,96 |
| Gemcitabine HCl | 0,27 | 0,23 | 0,86 |
| Daptomycin | 0,79 | 1,17 | 1,48 |
| Cytarabine | 0,26 | 0,26 | 1,03 |
| (-)-Tetramisole | 0,85 | 1,09 | 1,29 |
| Pacritinib (SB1518) | 0,58 | 0,44 | 0,76 |
| Fosinopril Sodium | 0,87 | 0,79 | 0,90 |
| Hydralazine HCl | 0,91 | 0,83 | 0,92 |
| Pimavanserin | 0,97 | 0,61 | 0,63 |
| Atosiban Acetate | 0,96 | 0,71 | 0,74 |
| Carboplatin | 0,81 | 0,54 | 0,66 |
| Dorzolamide HCl | 0,94 | 0,74 | 0,79 |
| L-Glutamine | 0,95 | 0,75 | 0,78 |
| Metformin HCl | 0,85 | 0,90 | 1,06 |
| Vorapaxar | 0,91 | 0,94 | 1,03 |
| Fudosteine | 0,90 | 0,74 | 0,81 |
| Oxacillin sodium monohydrate | 0,97 | 0,89 | 0,93 |
| Oclacitinib | 0,89 | 0,97 | 1,08 |
| Oxytocin (Syntocinon) | 0,94 | 0,98 | 1,04 |
| Pamidronate Disodium | 0,74 | 1,07 | 1,43 |
| Mizoribine | 0,89 | 0,94 | 1,06 |
| Nedaplatin | 0,35 | 0,30 | 0,85 |
| Ticlopidine HCl | 0,90 | 0,89 | 0,99 |
| Bardoxolone Methyl | 0,07 | 0,05 | 0,73 |
| Gabapentin | 0,91 | 0,77 | 0,84 |
| Neomycin sulfate | 0,94 | 0,83 | 0,88 |
| Bitopertin | 1,09 | 0,93 | 0,85 |
| Salmon Calcitonin Acetate | 1,02 | 0,87 | 0,86 |
| Gabapentin HCl | 0,96 | 0,87 | 0,90 |
| Polymyxin B sulphate | 0,86 | 0,82 | 0,96 |
| Penicillamine | 0,84 | 0,76 | 0,90 |
| Procarbazine HCl | 0,97 | 0,89 | 0,92 |
| Sotagliflozin (LX4211) | 1,14 | 1,19 | 1,04 |
| Donepezil HCl | 1,02 | 1,19 | 1,16 |
| Streptomycin sulfate | 1,05 | 1,28 | 1,22 |
| Ponesimod | 0,92 | 1,07 | 1,16 |
| GHRP-2 | 1,12 | 0,92 | 0,81 |
| Galanthamine HBr | 1,03 | 0,94 | 0,91 |
| LY2228820 | 0,74 | 0,72 | 0,97 |
| Etidronate | 1,01 | 1,02 | 1,01 |
| D-Cycloserine | 1,07 | 0,99 | 0,93 |
| Vancomycin HCl | 1,18 | 1,05 | 0,89 |
| Amikacin disulfate | 1,19 | 1,19 | 1,01 |
| Flavoxate HCl | 1,02 | 1,10 | 1,08 |
| Chloroquine Phosphate | 1,11 | 0,94 | 0,85 |
| Amifostine | 1,10 | 1,27 | 1,16 |
| Eprodisate (disodium) | 1,08 | 0,91 | 0,84 |
| Aprotinin | 1,02 | 0,94 | 0,92 |
| Calcium Levofolinate | 1,12 | 1,16 | 1,04 |
| (R)-baclofen | 1,03 | 0,99 | 0,96 |
| Colistin Sulfate | 0,97 | 0,99 | 1,02 |
| Ceftriaxone Sodium Trihydrate | 1,00 | 1,02 | 1,03 |
| Calcium Gluceptate | 1,01 | 1,04 | 1,04 |
| Pralidoxime (chloride) | 0,96 | 1,12 | 1,16 |
| Pemetrexed Disodium Hydrate | 0,44 | 0,65 | 1,48 |
| Hygromycin B | 0,82 | 0,80 | 0,98 |
| Amikacin hydrate | 1,11 | 0,94 | 0,85 |
| Gentamicin Sulfate | 0,96 | 1,01 | 1,05 |
| Nefopam HCl | 0,91 | 0,99 | 1,09 |
| Ceftazidime Pentahydrate | 0,94 | 0,98 | 1,04 |
| Eflornithine hydrochloride hydrate | 0,88 | 1,10 | 1,25 |
| Zanamivir | 1,06 | 1,02 | 0,96 |
| Tripelennamine HCl | 0,93 | 1,05 | 1,12 |
| Netilmicin Sulfate | 0,97 | 1,01 | 1,05 |
| Paromomycin Sulfate | 0,97 | 1,03 | 1,06 |
| Tolmetin Sodium | 0,82 | 0,98 | 1,19 |
| Glutathione | 0,86 | 1,06 | 1,23 |
| Solifenacin succinate | 1,23 | 1,19 | 0,96 |
| Ibandronate sodium | 1,02 | 0,96 | 0,94 |
| Hexamethonium Bromide | 0,91 | 0,91 | 1,00 |
| Minocycline HCl | 1,09 | 1,12 | 1,02 |
| Hydroxychloroquine Sulfate | 0,88 | 0,94 | 1,06 |
| L-Ornithine | 0,95 | 1,01 | 1,06 |
| Palonosetron HCl | 1,08 | 1,03 | 0,95 |
| Abacavir sulfate | 0,98 | 1,16 | 1,18 |
| Tetramisole HCl | 0,99 | 0,98 | 0,99 |
| Capreomycin Sulfate | 0,91 | 1,06 | 1,17 |
| Dihydrostreptomycin sulfate | 1,01 | 0,98 | 0,98 |
| Cefradine | 0,93 | 1,07 | 1,16 |
| Miltefosine | 1,03 | 1,15 | 1,12 |
| L-Arginine HCl (L-Arg) | 0,98 | 1,11 | 1,14 |
| Terbutaline Sulfate | 1,01 | 1,06 | 1,05 |
| Proflavine Hemisulfate | 0,16 | 0,19 | 1,15 |
| Sisomicin sulfate | 1,02 | 0,96 | 0,94 |
| Sildenafil | 0,91 | 0,95 | 1,05 |
| Danofloxacin Mesylate | 1,24 | 1,27 | 1,02 |
| Pemirolast potassium | 0,98 | 1,16 | 1,19 |
| Amprolium HCl | 1,15 | 1,14 | 1,00 |
| Sodium ascorbate | 1,05 | 1,05 | 1,00 |
| Antimonyl (potassium tartrate trihydrate) | 0,76 | 0,46 | 0,60 |
| Fosbretabulin (Combretastatin A4 Phosphate (CA4P)) Disodium | 0,11 | 0,13 | 1,16 |

**Supplementary Table 2.** Statistical differences of data presented in Figure 1B.

|  | **BIRINAPANT (µM)** | | | | | | | | | |
| --- | --- | --- | --- | --- | --- | --- | --- | --- | --- | --- |
|  | **0.005** | **0.01** | **0.025** | **0.05** | **0.1** | **0.25** | **0.5** | **1** | **2.5** | **5** |
| **H1299** vs.  **H1299-LKB1 KO 1** | ns | ns | ns | ns | p<0.01 | p<0.001 | p<0.0001 | p<0.0001 | p<0.0001 | p<0.0001 |

ns: not statistically significant

**Supplementary Table 3.** Statistical differences of data presented in Figures 2A and 2B.

|  | **Cell-Titer-Glo Assay**  **BIRINAPANT (µM)** | | | | | | | | | | | | | | | | | |
| --- | --- | --- | --- | --- | --- | --- | --- | --- | --- | --- | --- | --- | --- | --- | --- | --- | --- | --- |
|  | **0.005** | | **0.01** | | **0.025** | | **0.05** | | **0.1** | | **0.25** | | **0.5** | | **1** | | **2.5** | |
| **H1299** vs.  **H1299-LKB1 KO 1** | ns | | ns | | ns | | p<0.01 | | p<0.0001 | | p<0.0001 | | p<0.0001 | | p<0.0001 | | p<0.0001 | |
|  | **Sulforhodamine B Assay**  **BIRINAPANT (µM)** | | | | | | | | | | | | | | | | | |
|  | **0.005** | **0.01** | | **0.025** | | **0.05** | | **0.1** | | **0.25** | | **0.5** | | **1** | | **2.5** | | **5** |
| **H1299** vs.  **H1299-LKB1 KO 1** | ns | ns | | ns | | ns | | ns | | ns | | ns | | p<0.0001 | | p<0.0001 | | p<0.0001 |

ns: not statistically significant

**Supplementary Table 4.** Statistical differences of data presented in Figures 2C, 2D and 2E.

|  | **BIRINAPANT (µM)** | | | | | | | | | | | | | | | | | |
| --- | --- | --- | --- | --- | --- | --- | --- | --- | --- | --- | --- | --- | --- | --- | --- | --- | --- | --- |
|  | **0.005** | **0.01** | | **0.025** | | **0.05** | **0.1** | | **0.25** | | **0.5** | | | **1** | | **2.5** | | **5** |
| **H1299** vs.  **H1299-LKB1 KO 1** | ns | ns | | ns | | ns | p<0.001 | | p<0.0001 | | p<0.0001 | | | p<0.0001 | | p<0.0001 | | p<0.0001 |
| **H1299** vs.  **H1299-LKB1 KO 2** | ns | ns | | ns | | ns | ns | | ns | | p<0.001 | | | p<0.0001 | | p<0.0001 | | p<0.0001 |
| **H1299 LKB1 KO 1** vs.  **H1299-LKB1 KO 2** | ns | ns | | ns | | ns | ns | | ns | | ns | | | ns | | ns | | ns |
|  | **AT406 (µM)** | | | | | | | | | | | | | | | | | |
|  | **0.005** | | **0.01** | | **0.025** | | | **0.05** | | **0.1** | | **0.25** | **0.5** | | **1** | | **2.5** | |
| **H1299** vs.  **H1299-LKB1 KO 1** | ns | | p<0.01 | | ns | | | ns | | p<0.001 | | p<0.0001 | p<0.0001 | | p<0.0001 | | p<0.0001 | |
| **H1299** vs.  **H1299-LKB1 KO 2** | P<0.05 | | ns | | ns | | | ns | | p<0.01 | | p<0.0001 | p<0.0001 | | p<0.0001 | | p<0.0001 | |
| **H1299 LKB1 KO 1** vs.  **H1299-LKB1 KO 2** | p<0.01 | | p<0.05 | | ns | | | ns | | ns | | ns | p<0.05 | | p<0.001 | | ns | |
|  | **GDC152 (µM)** | | | | | | | | | | | | | | | | | |
|  | **0.005** | | **0.01** | | **0.025** | | | **0.05** | | **0.1** | | **0.25** | **0.5** | | **1** | | **2.5** | |
| **H1299** vs.  **H1299-LKB1 KO 1** | ns | | ns | | ns | | | p<0.01 | | p<0.0001 | | p<0.0001 | p<0.0001 | | p<0.0001 | | p<0.0001 | |
| **H1299** vs.  **H1299-LKB1 KO 2** | ns | | p<0.05 | | p<0.01 | | | p<0.001 | | p<0.0001 | | p<0.0001 | p<0.0001 | | p<0.0001 | | p<0.0001 | |
| **H1299 LKB1 KO 1** vs.  **H1299-LKB1 KO 2** | ns | | ns | | ns | | | ns | | ns | | p<0.0001 | ns | | ns | | ns | |

ns: not statistically significant

**Supplementary Table 5.** Statistical differences of data presented in Figure 2G.

|  | **BIRINAPANT (µM)** | | | | |
| --- | --- | --- | --- | --- | --- |
|  | **0.05** | **0.25** | **0.5** | **2.5** | **5** |
| **H1299 3D** vs.  **H1299-LKB1 KO 1 3D** | ns | ns | p<0.05 | p<0.0001 | p<0.0001 |

ns: not statistically significant

**Supplementary Table 6.** Statistical differences of data presented in Figure 3B.

|  | **BIRINAPANT (nM)** | | | |
| --- | --- | --- | --- | --- |
|  | **5** | **10** | **25** | **50** |
| **H1299 0h** vs.  **H1299-LKB1 KO 1 0h** | ns | ns | ns | ns |
| **H1299 24h** vs.  **H1299-LKB1 KO 1 24h** | ns | ns | ns | ns |
| **H1299 48h** vs.  **H1299-LKB1 KO 1 48h** | ns | ns | p<0.05 | p<0.05 |
| **H1299 72h** vs.  **H1299-LKB1 KO 1 72h** | ns | p<0.01 | p<0.0001 | p<0.0001 |

ns: not statistically significant

**Supplementary Table 7.** Statistical differences of data presented in Figure 3D.

|  | **BIRINAPANT (µM)** | | | |
| --- | --- | --- | --- | --- |
|  | **CTRL vs 0.2** | **CTRL vs 0.5** | **CTRL vs 1** | **CTRL vs 2** |
| **H1299 24h** | ns | ns | ns | ns |
| **H1299 48h** | ns | ns | ns | ns |
| **H1299-LKB1 KO 1 24h** | p<0.01 | p<0.0001 | p<0.0001 | p<0.0001 |
| **H1299-LKB1 KO 1 48h** | p<0.0001 | p<0.0001 | p<0.0001 | p<0.0001 |

ns: not statistically significant

**Supplementary Table 8.** Statistical differences of data presented in Figure 3F.

|  | **H1299**  **BIRINAPANT (µM)** | | | | | | |
| --- | --- | --- | --- | --- | --- | --- | --- |
|  | **0.05** | **0.1** | **0.25** | **0.5** | **1** | **2.5** | **5** |
| **BIRINAPANT** vs.  **BIRINAPANT + ZVAD 10 µM** | ns | ns | ns | ns | ns | ns | p<0.05 |
|  | **H1299-LKB1 KO 1**  **BIRINAPANT (µM)** | | | | | | |
|  | **0.05** | **0.1** | **0.25** | **0.5** | **1** | **2.5** | **5** |
| **BIRINAPANT** vs.  **BIRINAPANT + ZVAD 10 µM** | ns | ns | p<0.05 | p<0.0001 | p<0.0001 | p<0.0001 | p<0.0001 |

ns: not statistically significant

**Supplementary Table 9.** Statistical differences of data presented in Figure 5A.

|  | **K CLONE**  **BIRINAPANT (µM)** | | | | | | |
| --- | --- | --- | --- | --- | --- | --- | --- |
|  | **0.05** | **0.1** | **0.25** | **0.5** | **1** | **2.5** | **5** |
| **BIRINAPANT** vs.  **BIRINAPANT + RALIMETINIB 2 µM** | ns | ns | ns | ns | ns | p<0.05 | p<0.05 |
|  | **KL CLONE**  **BIRINAPANT (µM)** | | | | | | |
|  | **0.05** | **0.1** | **0.25** | **0.5** | **1** | **2.5** | **5** |
| **BIRINAPANT** vs.  **BIRINAPANT + RALIMETINIB 2 µM** | p<0.0001 | ns | p<0.05 | p<0.0001 | p<0.0001 | p<0.0001 | p<0.0001 |

ns: not statistically significant
